# Environmental Response to Root Secondary Metabolite Accumulation in Paeonia lactiflora: Insights from Rhizosphere Metabolism and Root-Associated Microbial Communities

**DOI:** 10.1128/spectrum.02800-22

**Published:** 2022-11-01

**Authors:** Xiao Sun, Xinke Zhang, Guoshuai Zhang, Yujing Miao, Tiexin Zeng, Min Zhang, Huihui Zhang, Li Zhang, Linfang Huang

**Affiliations:** a Key Lab of Chinese Medicine Resources Conservation, State Administration of Traditional Chinese Medicine of China, Institute of Medicinal Plant Development, Chinese Academy of Medical Sciences, Peking Union Medical College, Beijing, China; b College of Science, Sichuan Agricultural University, Ya’an, Sichuan, China; c State Key Laboratory of Southwestern Chinese Medicine Resources, Chengdu, Sichuan, China; CCG-UNAM

**Keywords:** plant-microbe associations, bacterial-fungal interactions, dispersal limitation, microbial interdomain networks, rhizosphere metabolome, *Paeonia lactiflora*

## Abstract

Paeonia lactiflora is a commercial crop with horticultural and medicinal value. Although interactions between plants and microbes are increasingly evident and considered to be drivers of ecosystem service, the regulatory relationship between microbial communities and the growth and root metabolites of *P. lactiflora* is less well known. Here, soil metabolomics indicated that carbohydrates and organic acids were enriched in the rhizosphere (RS) with higher diversity. Moreover, the variation of root-associated microbiotas between the bulk soil (BS) and the RS of *P. lactiflora* was investigated via 16S rRNA and internally transcribed spacer (ITS) amplicon sequencing. The RS displayed a low-diversity community dominated by copiotrophs, whereas the BS showed an oligotroph-dominated, high-diversity community. Hierarchical partitioning showed that cation exchange capacity (CEC) was the main factor affecting microbial community diversity. The null model and the dispersion niche continuum index (DNCI) suggested that stochastic processes (dispersal limitation) dominated the community assembly of both the RS and BS. The bacterial-fungal interkingdom networks illustrated that the RS possessed more complex and stable co-occurrence patterns. Meanwhile, positive link numbers and positive cohesion results revealed more cooperative relationships among microbes in the RS. Additionally, random forest model prediction and two partial least-squares path model (PLS-PM) analyses showed that the *P. lactiflora* root secondary metabolites were comprehensively impacted by soil water content (SWC), mean annual precipitation (MAP), pH (abiotic), and *Alternaria* (biotic). Collectively, this study provides a theoretical basis for screening the microbiome associated with the active components of *P. lactiflora*.

**IMPORTANCE** Determining the taxonomic and functional components of the rhizosphere microbiome, as well as how they differ from those of the bulk soil microbiome, is critical for manipulating them to improve plant growth performance and increase agricultural yields. Soil metabolic profiles can help enhance the understanding of rhizosphere exudates. Here, we explored the regulatory relationship across environmental variables (root-associated microbial communities and soil metabolism) in the accumulation of secondary metabolites of *P. lactiflora*. Overall, this work improves our knowledge of how the rhizosphere affects soil and microbial communities. These observations improve the understanding of plant-microbiome interactions and introduce new horizons for synthetic community investigations as well as the creation of microbiome technologies for agricultural sustainability.

## INTRODUCTION

Determining the composition and distribution of the rhizosphere (RS) microbes, as well as how they change from those of the bulk soil (BS) microbes, is critical for manipulating them to improve plant growth performance and increase agricultural yields (1, 2). The rhizosphere is an essential interface that allows plants and their soil environment to exchange resources (3). It is commonly acknowledged that plants can control rhizosphere microbial diversity and community by altering the composition of their root exudates (4). In other cases, the plant influences its microbiome by altering soil pH, decreasing competition for beneficial microorganisms, and providing an energy source, typically in the form of carbon-rich rhizodeposition (5). Plants utilize rhizobacterial assemblages in bulk soil pools to gain particular functional features required for plant fitness (2), and thus, the rhizosphere is a subset of the bulk soil microbe (6). The widespread consensus is that the diversity and composition of rhizosphere bacteria differ from those of bulk soil microbiota, which is linked to major changes in physicochemical parameters that drive niche differentiation (7). Aside from environmental variations across niches, the overall discrepancy in bulk soil and the rhizosphere is a significant driver of microbial distribution differences (8). The richness and composition of the rhizosphere microbes are influenced by plant species and soil properties (9). In addition, high-throughput sequencing of culture-independent marker genes (typically, 16S rRNA in bacteria and internal transcribed spacer [ITS] in fungi) has lately significantly expanded the repertory of soil microorganisms, and various studies have explored root-associated soil microbes, such as those for crops (10), Salvia miltiorrhiza (11), licorice (12), and citrus (13). Therefore, the composition and diversity of bacteria and fungi in *P. lactiflora* in bulk soil and the rhizosphere can be explored by amplicon sequencing.

Researching the microbiome on the soil-plant continuum is essential for investigating how these environmental factors respond to plant development and secondary metabolite (SM) synthesis (10). The effects of the microbiome on plant secondary metabolism of ginseng (14), licorice (15), etc., have been reported. Herbaceous peony (Paeonia lactiflora Pall.), a common garden plant in temperate climates (16) coming in a variety of flower types and colors, offers significant commercial benefits in the form of cut flowers, landscape architecture, and potted plants (17). Furthermore, the root of *P. lactiflora* is commonly used as a traditional medicine named Baishao, with analgesic and anti-inflammatory activities, to treat rheumatoid arthritis, hepatitis, and spasms (18). The root secondary metabolites of *P. lactiflora* are mainly represented by paeoniflorin (19). At present, the main research on *P. lactiflora* is focused on horticultural traits and pharmacological effects. However, research on the microbiome throughout the soil-plant continuum and how the microbiome interacts with *P. lactiflora* root secondary metabolites is limited. By considering the differences in soil metabolites, the different processes of microbial change in bulk soil and the rhizosphere can be improved (20). The metabolic profiles in root-associated soils contain a wide range of compounds that attract certain microbial species to develop intricate interactions with plants (21). Thus, chemical compounds in root-associated soil could influence soil microbes. Connecting chemicals to unique microbes can provide an understanding of the maintenance of microbial communities in root-associated soils (22). Overall, soil metabolites can disclose intricate molecular connections and metabolic functions in the soil microbe community, as well as providing a mechanism of evaluating soil function (23).

Here, this study collected two soil compartments (bulk soil [BS] and rhizosphere [RS]) from four primary areas of *P. lactiflora* production and performed amplicon sequencing, physicochemical property determination, and soil metabolism determination. Consequently, we presumed that (i) copiotrophic microbes are more common in the RS than in the BS (1), (ii) the diversity of the microbes in the BS is higher than that in the RS (10), (iii) the complexity of the bacterial-fungal interkingdom network in the rhizosphere is higher than that in the bulk soil, and therefore, the bacterial-fungal interkingdom network in the rhizosphere is more robust than that in the bulk soil (24), and (iv) the soil properties and microbial community of *P. lactiflora* would be partially correlated with root secondary metabolites (15). Specifically, our aims are to (i) detect the class and abundance differences of metabolites in *P. lactiflora* between the BS and RS, (ii) explore the compositional structure and dynamic ecological processes of bacterial and fungal communities between the BS and RS, and (iii) investigate the regulatory relationship between the microbiome and the root secondary metabolome of *P. lactiflora*.

## RESULTS AND DISCUSSION

### Variations of soil metabolomics between the BS and RS.

**FIG 1:**
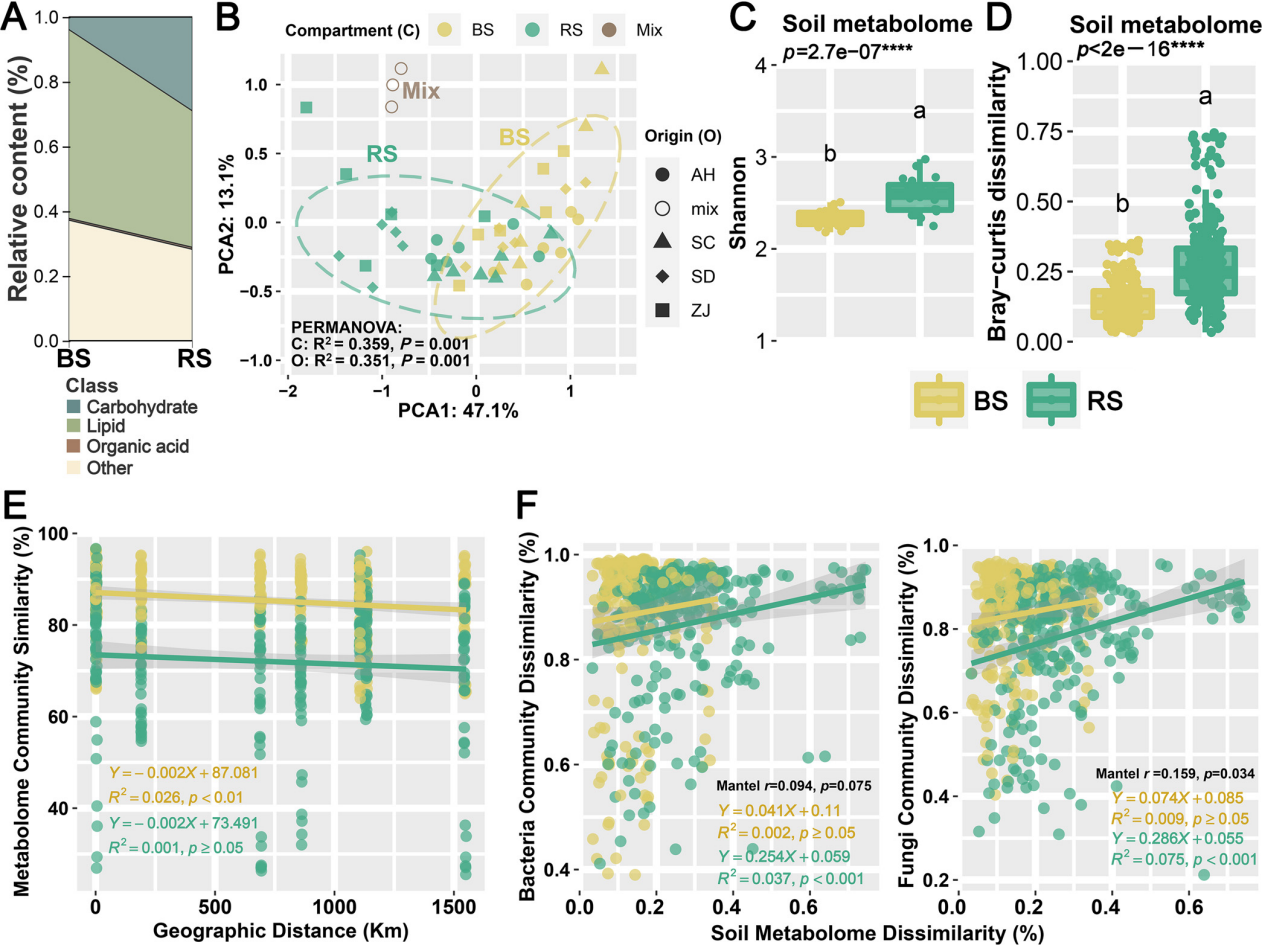
Soil metabolome profiles of the bulk soil (BS) and rhizosphere (RS) of *Paeonia lactiflora*. (A) The river plot shows the main class of soil metabolome. (B) Principal-component analysis (PCA) of soil metabolomics data. The degree of variation explained (*R*^2^) by the compartment (C) and origin (O) and the significance (*P* values) according to permutational multivariate analysis of variance (PERMANOVA) are provided. Mix, the balanced mixture of all samples (quality control); AH, Bozhou, Anhui; SC, Zhongjiang, Sichuan; SD, Heze, Shandong; ZJ, Pan’an, Zhejiang. (C) Alpha diversity (Shannon index) of soil metabolome. (D) Soil metabolome dissimilarity (Bray-Curtis distance) among all samples varied between the BS and RS. Different letters above the boxes indicate a significant difference. (E) Variations in soil metabolomics similarity (1–Bray-Curtis dissimilarity) with the gradients of the geographic distance. The rate of community turnover (slopes) across geographic distance and the fitting degree (*R*^2^) and significance (*P* values) of the linear fitting model are provided. (F) Correlations among bacterial (left) and fungal (right) community dissimilarity in the BS and RS with soil metabolomic dissimilarity are determined using the Mantel test. The correlation coefficients (*r*) and significance (*P* values) are provided. The *P* values are derived from one-tailed tests based on 999 permutations. The rate of bacterial and fungal community turnover (slopes) with the variation in soil metabolome and the fitting degree (*R*^2^) and significance (*P* values) of the linear fitting model are provided. The shaded region represents the 95% confidence limits of the regression estimates.

Through soil metabolomics, we can detect rhizosphere sediments secreted by plant roots, such as border cells, exudates, and mucilage, which influence the variation and activity of microorganisms in plant roots (25). To construct soil metabolic profiles of the bulk soil (BS) and the rhizosphere (RS), we performed untargeted metabolomic analysis of soil samples from 24 sampling sites in the four major *P. lactiflora* production areas in China (Bozhou in Anhui [AH], Zhongjiang in Sichuan [SC], Heze in Shandong [SD], and Pan’an in Zhejiang [ZJ]). Among all soil samples, 85 metabolites including carbohydrates (13, 15.29%), lipids (42, 49.41%), organic acids (5, 5.88%), and others (25, 29.41%) were discovered and classified (Fig. 1A and see Table S1 in the supplemental material). Notably, the relative abundance of organic acids (BS, 45.99%; RS, 54.01%) and carbohydrates (BS, 11.32%; RS, 88.68%) was higher in the RS than in the BS, while the relative abundance of lipids (BS, 56.89%; RS, 43.11%) was higher in the BS.

In general, plants have traditionally used rhizosphere secretions (including sugars, organic acids, amino acids, fatty acids, and secondary metabolites) to attract microorganisms from bulk soil to the rhizosphere for their own benefit, thereby shaping the rhizosphere microbial community (26). According to two groups of researchers (5, 27), the majority of the root exudates of various plants included organic acids and sugars, emphasizing the critical ecological function that these two classes of metabolites play in the recruitment or extinction of certain microbes in root-associated soils. The organic acids enriched in the rhizosphere in this study were mainly pipecolic acid and citrazinic acid (Table S1). Organic acids can increase the availability of phosphorus in soils, serve as metal chelators in the rhizosphere, act as electron donors in microbial anaerobic metabolism, and modulate enzyme activities (5). Previous studies have shown that under hypotonic stress, after cell decay, or during seed germination, pipecolic acid is released in the rhizosphere as an osmoprotectant for soil bacterial osmotically stressed cells (28).

Moreover, in root-associated soils, sugars are necessary for sustaining bacterial populations (20). Here, the d-threitol, d-fructose, d-(+)-talose, d-allose, d-glucitol, sucrose, etc., are the predominant carbohydrates enriched in the rhizosphere (Table S1). Carbohydrates and carbohydrate derivatives in general can be processed further by live cells to supply energy, and a variety of intermediates are engaged in the production of essential elements for life, including amino acids, fatty acids, and nucleic acids (29). Previous studies have shown that adding sugars from pine root exudates to soils affects the abundance and activity of various dominating taxa, like *Proteobacteria*, *Actinobacteria*, and *Firmicutes* (30). Some sugars in root exudates regulate rhizosphere bacteria’s chemotactic reactions, biofilm formation, and gene expression in addition to acting as carbon sources for microbes (31).

However, with the rise of new methodological approaches such as exometabolomics, microbial genomics, etc., root exudates were found to be highly complex mixtures containing metabolites from various chemical classes (32). Remarkably, some primary metabolites were detected by gas chromatography-mass spectrometry (GC-MS) in this study, among which lipids were the largest number detected, which is contrary to prior research (20). The relative abundance of benzoic acid, 2,4,7,9-tetramethyldec-5-yne-4,7-diol, methyl 2,6-dihydroxybenzoate, succinic acid, quinolinic acid, inositol, etc., was significantly upregulated in the rhizosphere. Previous studies have demonstrated complex subterranean interactions between plant roots and soil microbes through lipids (33). Clearly, plant lipids actively shape the microbiome that inhabits the rhizosphere and subsequently colonizes its root tissue. Furthermore, rhizosphere deposition and plant-microbe signaling are key subterranean processes in which lipids as chemical language are highly involved (34). Among known root exudates, benzoic acid is not rapidly and efficiently degraded by soil microorganisms, and its accumulation eventually alters soil microbial composition (35).

In addition, the abundance of soil metabolism in the RS was different from that in the BS. The diversity of the soil metabolites in the RS was significantly higher than that in BS (Fig. 1B to F, Supplemental Results, Table S1, and Fig. S1A and B). Notably, BS and RS soil metabolic classes were not particularly different, probably because the BS is also influenced by certain plants (samples were collected at 20 cm from the roots in a *P. lactiflora* farm field). This study’s results differed slightly from prior research on peppers (bulk soil was gathered 1 m away from the peppers) (36).

### The rhizosphere affects distribution and diversity of microbes in the BS and RS.

**FIG 2:**
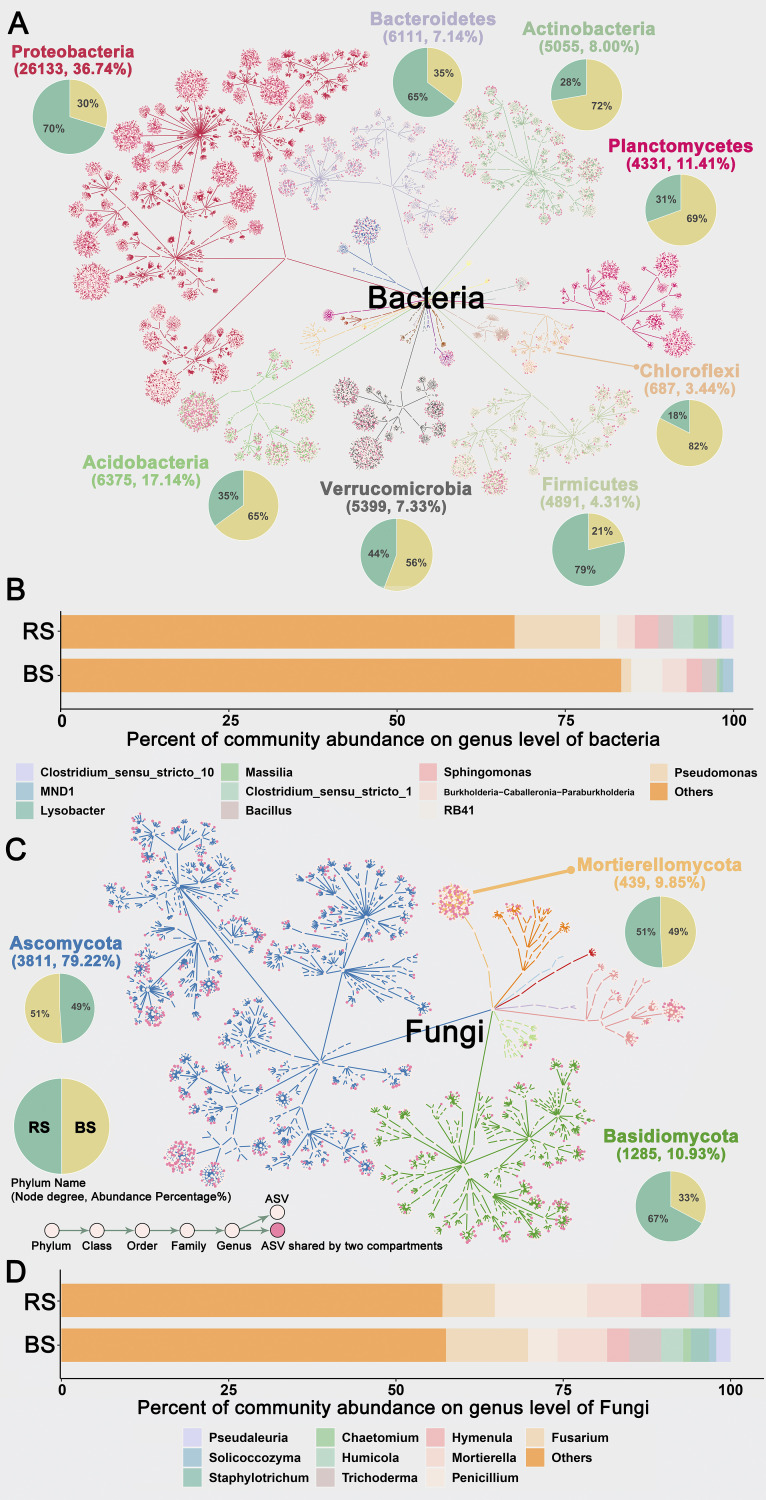
Taxonomic dendrograms of the detected microbial communities display the ASV distribution. Different colors represent different phylum levels. Each point represents the corresponding classification level, and the pink point displays the ASVs shared by the bulk soil and rhizosphere. The pie chart shows the proportion of this phylum in the two compartments of soil microbial communities. (A) Bacteria. (C) Fungi. (B) Bacterial community (top 10 genera in relative abundance). (D) Fungal genus community (top 10 genera in relative abundance). BS, bulk soil; RS, rhizosphere.

Bulk soil (BS) can be regarded as a “microbial seed bank” from which rhizosphere (RS) microorganisms are recruited, and hence, the diversity of rhizosphere microorganisms is reduced (1). To understand the differences in microbial composition between the BS and RS, statistical analyses were performed on the annotated phyla and genera. A taxonomic dendrogram (Fig. 2A) revealed the most numerous types of amplicon sequence variant (ASV) classifications of bacteria at the phylum level: *Proteobacteria* (36.74%), *Acidobacteria* (17.14%), and *Bacteroidetes* (7.14%). Among the bacterial phyla in the RS, *Bacteroidetes* (BS, 35.27%; RS, 64.73%), *Firmicutes* (BS, 21.26%; RS, 78.74%), and *Proteobacteria* (BS, 29.79%; RS, 70.21%) were enriched in the RS. Normally, these phyla are widely suited to C-rich environments (typically found in the rhizosphere) for high metabolic activity, fast growth, and reproduction and thus are sometimes referred to as copiotrophs (3, 21). Conversely, *Chloroflexi* (BS, 82%; RS, 18%), *Acidobacteria* (BS, 64.98%; RS, 35.02%), and *Actinobacteria* (BS, 72.24%; RS, 27.76%) were enriched in the BS. Except for *Actinobacteria*, most of the other phyla (*Acidobacteria*, *Nitrospirae*, *Gemmatimonadota*, and *Chloroflexi*) enriched in the BS were defined as oligotrophs (1). Numerous studies demonstrate that oligotrophs develop more slowly under resource-poor conditions while copiotrophs grow more quickly (37). While oligotrophs effectively exploit resources at the expense of growth rate, copiotrophs grow more quickly and depend on the availability of resources (38). It is worth noting that some phyla are not absolutely copiotrophs or oligotrophs. Both copiotrophs and oligotrophs were present in the *Bacteroidetes* and *Proteobacteria* phyla as reported in previous studies (39). Therefore, further exploration is needed according to a more rigorous physiological ability and at a more refined taxon level. The relative abundance of bacterial genera showed that the abundance of genera Pseudomonas (BS, 10.48%; RS, 89.52%), *Sphingomonas* (BS, 39.65%; RS, 60.35%), and *Massilia* (BS, 14.01%; RS, 85.99%) in the RS was higher than that in the BS (Fig. 2B).

For fungi (Fig. 2C), the most abundant phyla were Ascomycota (79.22%), Basidiomycota (10.93%), and Mortierellomycota (9.85%), which is found to be a common pattern in soils globally (40). Only the abundance of Basidiomycota was higher in the RS (BS, 33%; RS, 67%). The subkingdom Dikarya, sometimes known as the “higher fungi,” is made up of the two major divisions Basidiomycota and Ascomycota, which together make up the kingdom Fungi (41). The phylum Glomeromycota is entirely comprised of photoautotroph symbiotic fungi, such as arbuscular mycorrhizal fungi (AMF) (42). However, the relative abundance of Glomeromycota in the BS is higher than that in the RS, but the relative abundance of this phylum was low for the whole. The relative abundance of bacterial genera showed that the abundance of genera Pseudomonas (BS, 10.48%; RS, 89.52%), *Sphingomonas* (BS, 39.65%; RS, 60.35%), and *Massilia* (BS, 14.01%; RS, 85.99%) in the RS was higher than that in the BS (Fig. 2B). The relative abundance of fungal genera showed that the abundance of genera *Penicillium* (BS, 24.24%; RS, 75.76%), *Hymenula* (BS, 32.01%; RS, 67.99%), and *Chaetomium* (BS, 36.40%; RS, 63.60%) in the RS was higher than that in the BS (Fig. 2D). Indicator analysis (Table S3) showed that bacterial indicators were mainly enriched in the phylum *Proteobacteria*, while those in fungi were mainly in the phyla Ascomycota (BS) and Basidiomycota (RS).

**FIG 3:**
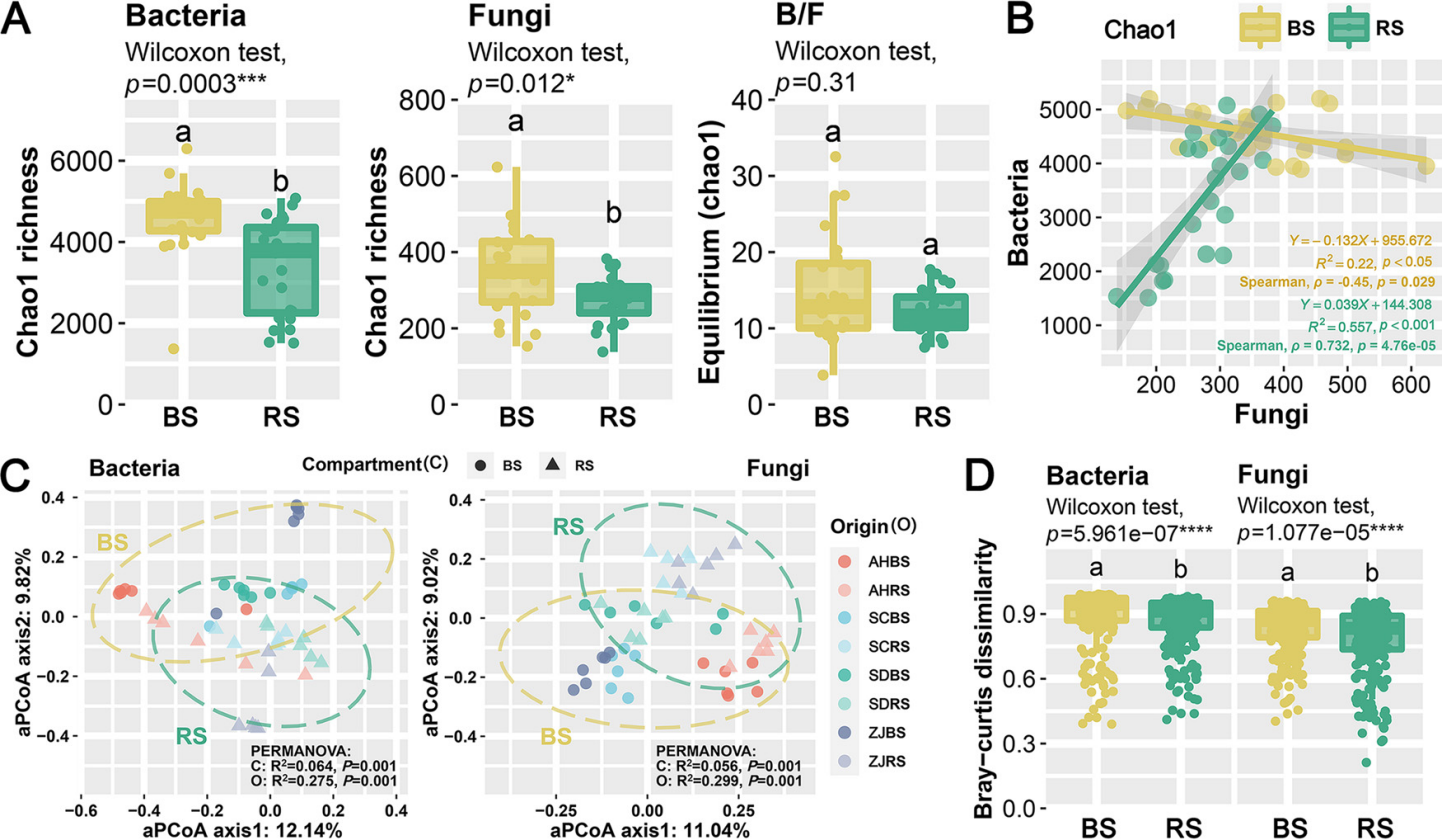
The diversity and distribution patterns of soil microbial communities of *P. lactiflora* in the bulk soil (BS) and rhizosphere (RS). (A) Alpha diversity of bacterial (left) and fungal (middle) communities. The ratio of alpha diversity of bacteria and fungi (B/F) represents equilibrium (right), which reflects the degree of microbial equilibrium. (B) The linear relationships and Spearman correlations between the alpha diversity indices of soil bacterial and fungal communities in the rhizosphere and bulk soil. The fitting degree (*R*^2^) and significance (*P* values) of the linear fitting model are provided. (C) Adjusted principal-coordinate analysis (aPCoA) ordinations based on Bray-Curtis distance matrices describing the distribution patterns of bacterial and fungal communities. The degree of variation explained (*R*^2^) by the compartment (C) and origin (O) and the significance (*P* values) according to permutational multivariate analysis of variance (PERMANOVA) are provided. AH, Bozhou, Anhui; SC, Zhongjiang, Sichuan; SD, Heze, Shandong; ZJ: Pan’an, Zhejiang. (D) Microbial community dissimilarity among all samples varied across the BS and RS. Different letters above the boxes indicate a significant difference determined by the nonparametric Wilcoxon rank-sum test.

Community diversity is another statistic frequently used in soil microbiome investigations, where greater diversity is typically regarded as advantageous for the soils as a whole (43). The alpha diversity index was calculated to quantify the species richness of microbial communities, and the results showed that the Chao1 index was significantly higher in the BS than in the RS, and the bacterial community was higher than the fungal community (Fig. 3A). This result is consistent with the research results of microbiome analysis such as those for corn (10) and licorice (15). In contrast, the diversity of soil metabolism was greater in the RS than in the BS (Fig. 1C and D); therefore, subsequent studies can be combined with new technologies such as microbial genomics and epimetabolomics to understand the metabolic niches in the rhizosphere microbiome more clearly. The results of latitudinal diversity were shown in Fig. S2A. The key environmental factors influencing microbial diversity were determined using Spearman correlation (Fig. S2E).

Principal-coordinate analysis (PCoA) is used to describe the differences between samples, and Shi et al. (44) developed an adjusted principal-coordinate analysis (aPCoA) based on PCoA, by adjusting for covariates to exclude their effects. aPCoA ordinations and permutational multivariate analysis of variance (PERMANOVA) (Fig. 3C) revealed that origin site (bacteria, *R*^2^ = 27.5%, *P* = 0.001; fungi, *R*^2^ = 29.9%, *P* = 0.001) explained the majority of the variation in bacterial and fungal communities, followed by compartment niche (bacteria, *R*^2^ = 6.4%, *P* = 0.001; fungi, *R*^2^ = 5.6%, *P* = 0.001). Moreover, microbial community dissimilarity was significantly lower in the RS than in the BS (Fig. 2D), indicating a homogenizing effect of plants on community structure (6). The selection effects of the rhizosphere on particular species are primarily responsible for the variations in the bacterial and fungal community between the RS and BS (45). These results provide potential evidence for inferring rhizosphere effects (46). The beta diversity pattern includes two independent components: turnover (species replacement) and nestedness (species loss) (47). The diversity decomposition analyses revealed that species replacement processes dominated microbe compositional dissimilarities (contributing 79.17% and 76.21% for bacterial and fungal diversity, respectively), whereas richness difference processes attributed only 20.83% and 23.79% on average (Table S4). For both bacteria and fungi, species replacement processes were significantly higher in the BS than in the RS. The richness difference processes of bacteria in the BS were significantly lower than in the RS, while the fungi in the BS were significantly higher than in the RS (Fig. S3). The spatial turnover of microbes was typically consistent with the distance-decay trend at the spatial scale (Fig. S2E).

Bacteria and fungi in the same region have large differences in cell structure and ecological niche but are also closely related. Therefore, it is necessary to comprehensively understand the relationship between bacteria and fungi in the BS and RS (Fig. 3B, Supplemental Results, Fig. S2B to D, and Table S5).

### Community assembly was governed by stochastic processes primarily belonging to dispersal limitation.

**FIG 4:**
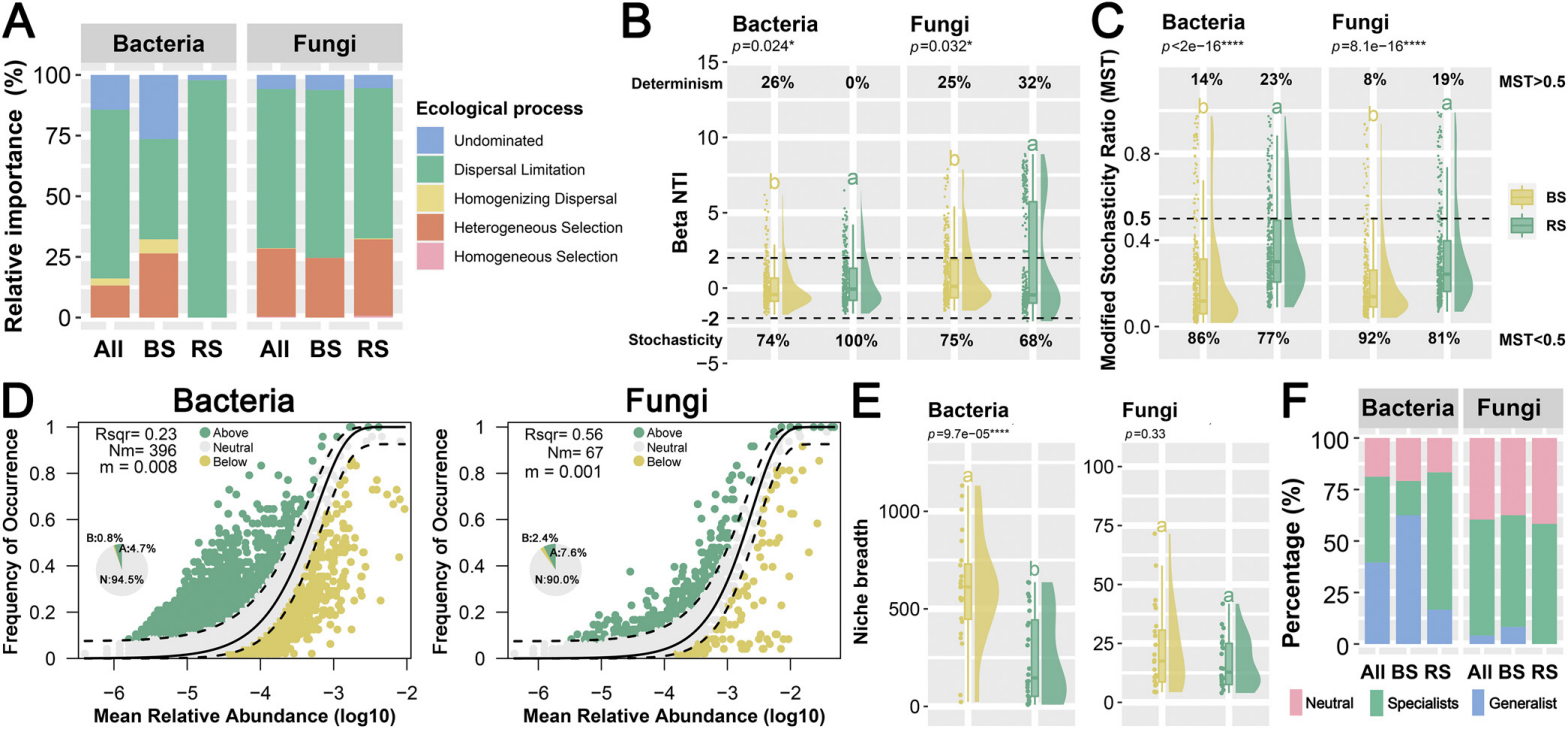
The ecological processes of soil bacterial and fungal communities. (A) The relative importance of five ecological processes (heterogeneous selection, βNTI < −2; homogeneous selection, βNTI > + 2; dispersal limitation, |βNTI| < 2 and RC_Bray_ >0.95; homogenizing dispersal, |βNTI| < 2 and RC_Bray_ < −0.95; and undominated, |βNTI| < 2 and |RC_Bray_| < 0.95) between the rhizosphere and bulk soil based on the beta nearest taxon index (βNTI) and Bray-Curtis-based Raup-Crick index (RC_Bray_). (B) Positive and negative βNTI values indicate greater and less than the expected turnover of the phylogenetic composition, respectively. The horizontal dashed line (values above −2 or below +2 are statistically significant) shows the 95% confidence intervals around the expectation under neutral community assembly. The proportion of the stochastic processes (|βNTI| < 2) and deterministic processes (|βNTI| > 2) is shown in percentage numbers. Different letters above the boxes indicate a significant difference. (C) Modified stochasticity ratio (MST) of bacterial and fungal communities of the bulk soil and rhizosphere. Different letters above the boxes indicate a significant difference. (D) The fit of the neutral community model (NCM) of microbial community assembly. *R*^2^ indicates the fit of this model. *Nm* is the product of metacommunity size (*N*) and migration rate (*m*), which quantifies the estimate of dispersal between communities and determines the correlation between frequency of occurrence and relative regional abundance. The solid line indicates the best fit to the NCM, and the dashed lines represent 95% confidence intervals around the model prediction. The pie charts depict the ratio of “above” taxa (ASVs that occur more frequently than predicted by the model are shown in green), “neutral” taxa (ASVs that occur within prediction are shown in gray), and “below” taxa (ASVs that occur less frequently than predicted are shown in yellow). (E) Distribution of niche breadth indices for the bacterial and fungal communities. (F) Distribution of generalized and specialized species in the bacterial and fungal communities of the bulk soil and rhizosphere. The occurrences of ASVs generated by simulating 1,000 permutations were determined using the EcolUtils R package. Generalist species have wider fundamental niches than specialists. The ASV is considered generalist or specialist based on whether the observed occurrence exceeded the upper 95% confidence interval or fell below the lower 95% confidence interval, and the ASVs were considered neutral taxa if the observed niche breadth was within the 95% confidence interval range.

To gain insight into the microbiome, it is essential to understand the ecological mechanisms that shape the communities (48). Previous studies (49, 50) have indicated that the mechanisms of community assembly can be classified into stochastic processes based on the neutral hypothesis (dispersal limitation, etc.) and deterministic processes based on the niche theory (environmental filtration and biotic interactions). The results of beta nearest taxon index (βNTI) and RC_Bray_ (Raup-Crick) demonstrated that stochastic processes dominate the assembly of microbial communities in the BS and RS, especially dispersal limitation (Fig. 4A and B). Meanwhile, the deterministic process focused on heterogeneous selection (Fig. 4A). Heterogeneous selection is selection under heterogeneous abiotic and biotic environmental circumstances, resulting in more-dissimilar community structures (51). For bacteria, the RS had a larger relative contribution of stochastic processes mostly related to dispersal limitation (~97.8%) than the BS (~41.3%). Likewise in fungi, dispersal limitation occupied the highest proportion in the stochastic processes of the BS (69.2%) and RS (62.0%). Random fluctuations in the relative abundance of species are principally represented by stochastic processes, which include random birth, death, and dispersion occurrences (52). Previous studies have found that dispersal limitation plays an important role in driving beta diversity at small and medium sample sizes (53). It is important to note that the dispersion limit cannot be regarded as the only indicator of a stochastic process because dispersion can be either deterministic or stochastic (51). For results of other analytical methods (modified stochastic ratio [MST] [Fig. 4C] and dispersal niche continuum index [DNCI] [Table S6]) to quantify community assembly, please see the Supplemental Results for details.

The Sloan neutral community model (NCM) elucidated the possible role of stochastic events in the formation of bacterial and fungal communities in the BS and RS (Fig. 4D and Fig. S4). The relative contribution of stochastic processes explained 23% and 56% of the community variance for the total bacterial and fungal communities, respectively. This indicated that the overall fungal community was more impacted by stochastic processes than was the total bacterial community. In contrast, the entire fungal community’s migration rate (0.001) was lower than that of the total bacterium community (0.008), indicating that species dispersion was more limited in the total fungal community (Fig. 4D). For two soil compartments (Fig. S4), the migration rate of the RS (bacteria, 0.009693; fungi, 0.0022) was higher than that of the BS (bacteria, 0.009684; fungi, 0.0016). The microbial community immigration rate in the RS was higher than that in the BS, indicating that the dispersing capacity of most microbial taxa in the RS was greater than that in the BS. Furthermore, according to the neutral model, stochastic processes should be taken into account even in a highly selective environment like the rhizosphere since dispersion restriction is important in the construction of microbial communities (Fig. 4D). The *R*^2^ of fungi in the BS was higher than in the RS, suggesting that the aggregation of fungal communities in the BS was more driven by stochastic processes than was that in the RS. In general, because of the selected role of root excretions (54), deterministic processes account for a greater percentage of rhizosphere microbe assembly than that in bulk soil, which agreed with the results for fungi.

To investigate the relative role of deterministic and stochastic mechanisms in microbial community assembly, community-level habitat niche breadths (*Bcom*) were estimated (Fig. 4E). Both in the BS and in the RS, bacteria were found to have a considerably higher *Bcom* value than fungus (*P* < 0.001), and the niche breadth of the BS was larger than that of the RS but not significantly so. According to a prior study, species with larger niche breadths may exhibit more metabolic adaptability and be less subject to deterministic processes (55). However, the *R*^2^ (NCM) of bacteria in the RS was lower than that in the BS, which may be related to sampling scale, historical factors, random drift, and other factors (53). Based on the size-plasticity theory (also known as the “body size effect”), ecological determinism would rise with organism size since smaller organisms (such as bacteria) are less environmentally filtered than bigger organisms (56). Conversely, the *R*^2^ of the neutral model was higher for fungi than for bacteria, suggesting a greater contribution of stochastic processes to fungi. In addition, the niche breadth index was used to distinguish generalists and specialists based on whether it was more or less than simulated chance, respectively (Fig. 4F). In the BS, generalists have the largest proportion of bacteria (63%), while specialists have the largest proportion of fungi (54%). In the RS, specialists have the largest proportion of both bacteria (67%) and fungi (58%). Furthermore, deterministic processes have a greater impact on environment specialists with restricted ecological niche widths than on generalists with broad ecological niche widths (57). Species were divided according to the simulated null distribution, with the highest proportion of specialists in the RS, which also appeared to be related to rhizosphere effects.

Distance-decay relationships (DDRs) are known to be affected by both deterministic and stochastic processes (58). The distance-decay pattern, which predicts decreased community resemblance with rising geographic distance, has been intensively studied in the biogeography of microbiota (59). The similarity of bacterial and fungal communities in the BS and RS decreased with increasing geographical distance, indicating that significant DDR patterns were present in both bacteria and fungi (Fig. S2C). The distance-decay pattern was established using four ecological processes: mutation, drift, dispersion, and selection. The slope of the distance-decay curve is increased by selection and drift, but this connection is weakened by dispersal (60). For bacteria, the DDR slope of the RS is steeper than that of the BS, which implies that historical factors (such as geographic isolation) have a stronger impact on the rhizosphere (45). In contrast, the DDR slope of the RS in fungus was lower than that of the BS, which might be attributed to the function of environmental choice in fungal community spatial variation (61).

Deterministic processes include the selection of abiotic environmental factors (environmental filtering) and the mutual antagonism and synergy between species. Furthermore, soil physicochemical characteristics such as pH, wetness, and soil characteristics can influence root-associated microbial communities (62). The results of the Mantel test, canonical correspondence analysis (CCA), and hierarchical partitioning analysis indicated that the cation exchange capacity (CEC) significantly affected the microbial community diversity of the BS and RS (Supplemental Results, Tables S5 and S7, and Fig. S5 and S6). Notably, the results of the hierarchical partitioning indicated that environmental variables had a quite low interpretation rate (bacteria, 6.3%; fungi, 8.1%) and a large level of the unexplained component (bacteria, 93.7%; fungi, 91.9%). Correspondingly, it is speculated that the deterministic process is dominated by species-to-species interactions.

### The complexity and stability of the interkingdom network of the RS were higher than those of the network of the BS.

**FIG 5:**
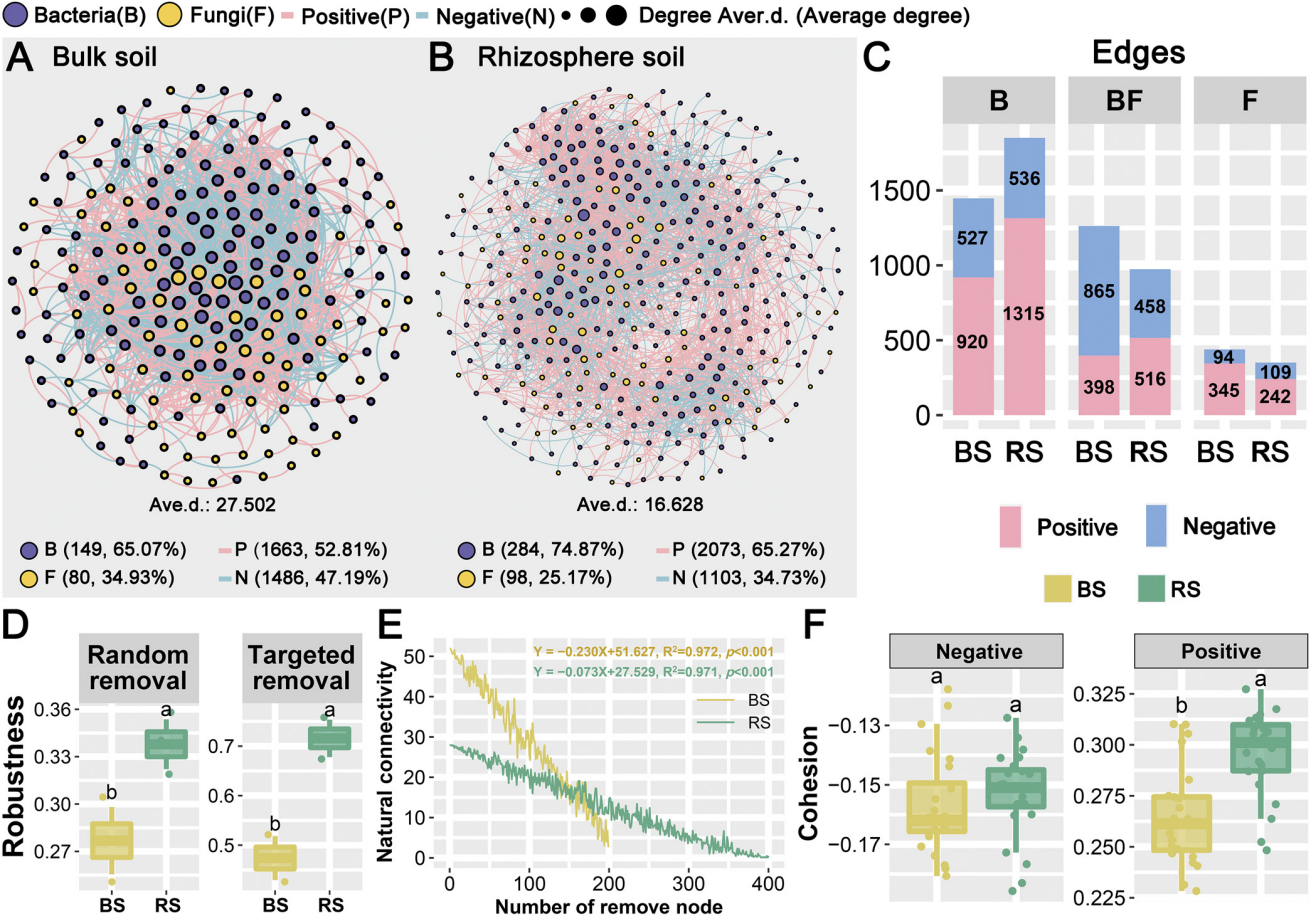
The complexity and stability of soil bacterial-fungal interkingdom networks. (A and B) Co-occurrence patterns of bacterial-fungal interkingdom networks in the bulk soil (A) and rhizosphere (B) of *P. lactiflora*. (C) The bar graph shows the proportion of inter- and intra-kingdom edges of positive (pink) or negative (blue) correlations in the bulk soil and rhizosphere network. B, bacterial intrakingdom; F, fungal intrakingdom; BF, bacterial-fungal interkingdom association. (D) Robustness was measured as the proportion of taxa that remained with 50% of the taxa randomly removed from each of the bacterial-fungal interkingdom networks. Robustness was also measured as the proportion of taxa that remained with 10 module hubs removed from each of the bacterial-fungal interkingdom networks. *, *P* value < 0.05; **, *P* value < 0.01. (E) The robustness of the microbial networks is based on natural connectivity. (F) Cohesion of bacterial-fungal interkingdom network communities in the bulk soil and rhizosphere. Positive cohesion is generated by pairwise positive correlation, which can reflect the degree of cooperative behavior in the sample, whereas negative cohesion can represent the degree of competitive behavior among community members. Different letters above the boxes indicate a significant difference.

Cooccurrence patterns are frequent and essential to comprehending the composition of microbes (14). Interkingdom network analysis was performed to assess the impact of bacterial-fungal interkingdom interactions between the BS and RS (Fig. 5A and B and Fig. S7A and B). To determine the difference in the complexity of the microbial network between the BS and RS, the changes in the topological parameters (Table S8) of the microbial network were calculated. The cooccurrence network of the BS consisted of 229 nodes (bacteria, 149; fungi, 80) and 3,149 edges (positive, 1,663; negative, 1,486), whereas those of the RS consisted of 382 nodes (bacteria, 284; fungi, 98) and 3,176 edges (positive, 2,073; negative, 1,103). This was consistent with the complexity of the bipartite network in the BS and RS. Specifically, the BS had higher network connectivity (i.e., average degree) than the RS. The above results showed that the complexity of the RS bacterial-fungal interkingdom network was stronger than that of the BS network, which was similar to the results for licorice (15). However, in the microbial network results of different developmental stages of maize, the network complexity of the BS and RS at different stages is different (10). The samples in this study were collected at the mature stage of *P. lactiflora*, and the next step in the study of network complexity may require sampling at different periods. The intricate linkages seen in the RS networks might be attributed to more frequent nutrient exchanges and more stable network topologies that can withstand external disturbance (15). The links for bacteria and fungi themselves were mostly positive. In contrast, the association between bacteria and fungi was mainly negative in the BS, while positive links were higher than negative correlations in the RS (Fig. 5C). In this study, the positive correlation between microbes was stronger in the RS than in the BS, indicating that the rhizosphere’s microbes may engage in substantial mutualistic interactions (63).

Based on natural connectivity analysis, the highest robustness was observed in the RS network. Meanwhile, species extinction (resilience to node loss) of the interkingdom network was simulated to compute the robustness of the network. The RS network was more stable (*P* < 0.001) than the BS network under either random node loss or targeted removal of module hubs (Fig. 5D). The network vulnerability (the highest reduction in network efficiency when a single node was removed from the network) was likewise lower in the RS (0.01072515) than in the BS (0.01960996), indicating that RS networks are more resistant. According to the natural connectivity analysis, the BS network had more natural connectedness than the RS network when fewer nodes were randomly deleted, and the RS network had higher natural connectivity when more nodes were removed (Fig. 5E). Studies of network stability with warming control show that network stability rises with network complexity, particularly relative modularity, which is congruent with macroecological data. Simultaneously, our findings confirm MacArthur’s claim that ecological complexity contributes to stability (24). Additionally, the level of network complexity was assessed using a newly constructed metric known as cohesion. Positive cohesion can define the extent of cooperative conduct in the community, whereas negative cohesion can show the level of competitive activity in the community (24). Positive cohesion of the RS network was significantly higher than that of the BS network, while negative cohesion of the RS network was marginally but not significantly higher than that of the BS network (Fig. 5F). Since rhizosphere microbes are driven to homogeneous selection by root exudates, more cooperative (positive) linkages in the rhizosphere might exist (54).

Keystone nodes that contribute significantly to forming the network structure were discovered in all interkingdom networks based on within-module connectivity (*Z_i_*) and among-module connectivity (*P_i_*), along with 10 module hubs, 64 connectors, and 7 hubs (Fig. S7C). In the BS, network hubs contained only 6 fungi, and connectors contained 38 bacteria and 17 fungi. In the RS, network hubs had 1 fungus, connectors included 7 bacteria and 2 fungi, and module hubs had only 10 fungi (Table S8).

### Integrated modulations of *P. lactiflora* root secondary metabolites.

**FIG 6:**
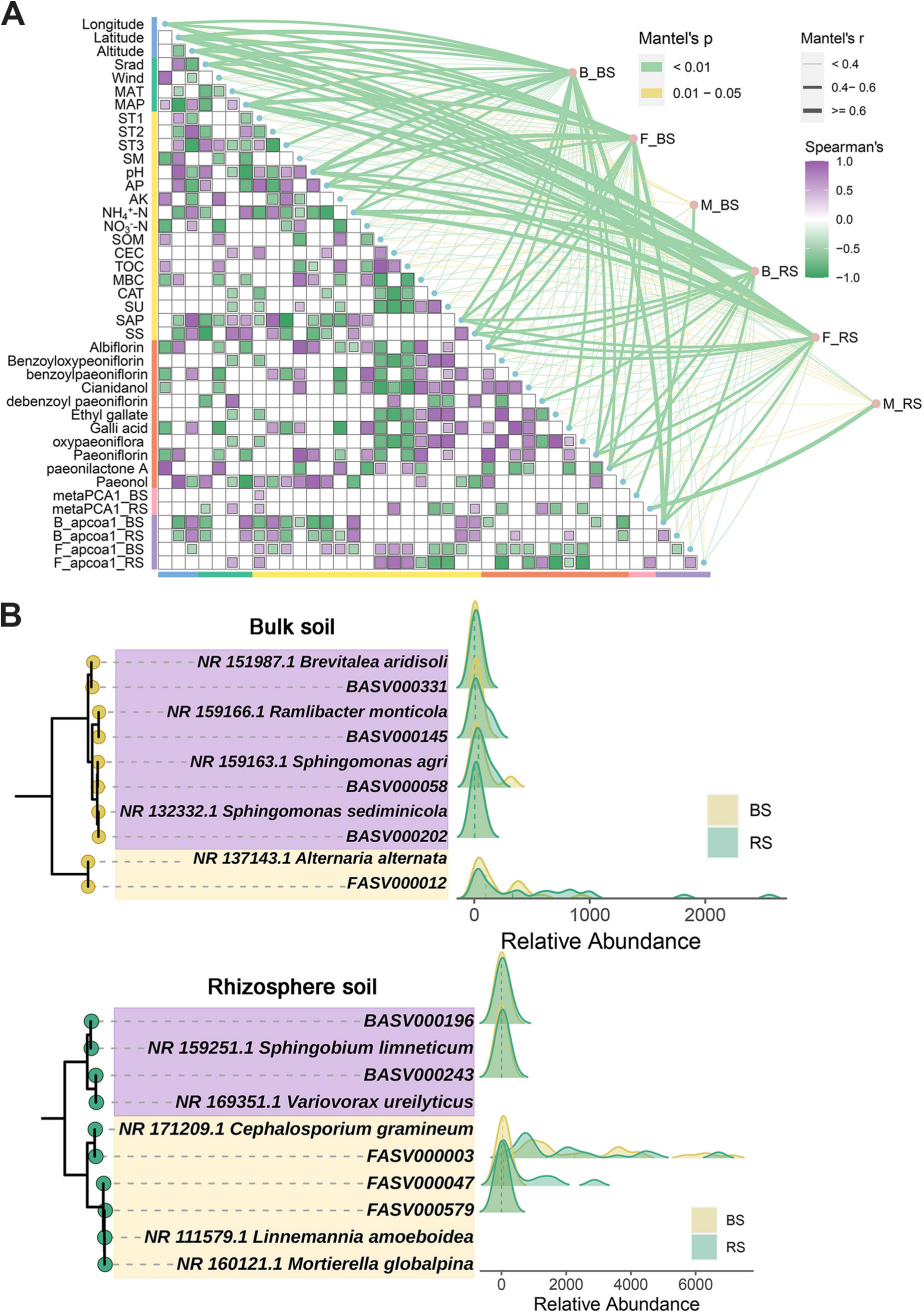
Interactions among environment, soil metabolites, root secondary metabolites, and microbiotas. (A) Correlations of the microbial community and soil metabolite structures (Bray-Curtis distance) with geographical variables (blue), climatic characteristics (green), edaphic properties (yellow), root secondary metabolites (orange), soil metabolites (pink), and microbial communities (purple) in the bulk soil and rhizosphere determined using the Mantel test. The color of the line represents the significance of the differences (*P* values). The size of the line represents the size of the correlation coefficients (Mantel’s *r*). The pairwise correlations of these variables are shown with a color gradient representing the Spearman correlation coefficient. B/F_BS/RS, bacteria/fungi in the bulk soil/rhizosphere; M_BS/RS, soil metabolites in the bulk soil/rhizosphere. Geographical variables include longitude, latitude, and altitude. Climatic variables include solar radiation (Srad), wind speed (wind), mean annual temperature (MAT), and mean annual precipitation (MAP). Refer to Tables S13 in the supplemental material for specific variables of soil and climate. Soil metabolites involving metaPCA1_BS and metaPCA1_RS represent the first axes of principal-component analysis (PCA) of soil metabolites in the bulk soil and rhizosphere. Microbial communities containing B_apcoa1_BS, B_apcoa1_RS, F_apcoa1_BS, and F_apcoa1_RS represent the first axes of adjusted principal-coordinate analysis (aPCoA) of bacterial and fungal communities in the bulk soil and rhizosphere based on Bray-Curtis distances. (B) Identification of key ASVs associated with paeoniflorin content predicted by random forest model. Ridgeline plots show the relative abundance of key ASVs in the bulk soil and rhizosphere. The key ASVs and the sequences obtained from the BLAST search results on NCBI are constructed using the maximum likelihood method to construct a phylogenetic tree.

Secondary metabolites (SMs) of *P. lactiflora* (Table S9) are the material basis for its clinical efficacy and an important component of *P. lactiflora* defense against pathogenic attack and environmental stress (64). Abiotic and biotic environments that are constantly changing have an impact on the appropriate synthesis and accumulation of SMs, which are tightly regulated in both space and time (65). The *P. lactiflora* root secondary metabolites were influenced by both abiotic ((location, soil, and climate) and biotic (diversity and composition of microbes) variables. Soil water content (SWC), mean annual precipitation (MAP), and pH influenced the secondary metabolites of *P. lactiflora* roots (Fig. 6A, Supplemental Results, Table S10, and Fig. S8).

Plant microbiomes have been proven in many studies (66) to impact host plant productivity of essential medicinal components such as alkaloids, steroids, terpenoids, etc. For example, studies have shown that plant-microbiome interactions can increase the biomass yield of *Salvia miltiorrhiza* and affect tanshinone production. The unique seed-associated microbiome (*Pantoea*, Pseudomonas, *Sphingomonas*, and *Dothideomycetes*) of *S. miltiorrhiza* contains a gene pool associated with the synthesis of tetraterpene backbones and other compounds, thereby providing additional metabolic capacity to the host plant (11). In addition, several studies have reported the beneficial effects of arbuscular mycorrhizal symbiosis on plant growth and secondary metabolite accumulation in licorice roots (12). The microbiome related to the secondary metabolism of *P. lactiflora* was screened, which provided a theoretical basis for the subsequent isolation and culture of bacteria and fungi. The random forest regression model analysis was performed to identify microbial genera (bacteria-BS, *Rhodoplanes* and *Pseudoxanthomonas*; bacteria-RS, *Erwinia* and *Sphingobium*; fungi-BS, *Apiotrichum* and *Solicoccozyma*; fungi-RS, *Alternaria* and *Vishniacozyma*) affecting paeoniflorin (Supplemental Results and Fig. S9).

Weighted gene coexpression network analysis (WGCNA) was used to compress the data of two soil compartments and found the brown (BS) and pink (RS) modules, which were highly linked to the root secondary metabolites, in order to perform dimensionality reduction analysis on the complex bacterial and fungal abundance data (Fig. S10A and B). Overall, the random forest model and cooccurrence network analysis identified the hub ASVs related to paeoniflorin (Fig. S10C and D). In order to accurately identify the taxa of the key ASVs, NCBI BLAST searching was performed, and the highest-alignment sequences were selected to construct maximum likelihood trees (ML trees) with the key ASVs in the BS and RS (Fig. 6B and Table S11). The key bacteria identified in the BS were Sphingomonas agri (BASV000058), Ramlibacter monticola (BASV000145), Sphingomonas sediminicola (BASV000202), and Brevitalea aridisoli (BASV000331), and the fungus was Alternaria alternata (FASV000012). In the RS, the key bacteria detected were Sphingobium limneticum (BASV000196) and Variovorax ureilyticus (BASV000243), and the fungi were Cephalosporium gramineum (FASV000003), Mortierella globalpina (FASV000047), and Linnemannia amoeboidea (FASV000579). WGCNA screening found that Alternaria tenuissima and A. alternata were significantly correlated with paeoniflorin content. *A. tenuissima* is widespread, occurring on a wide range of different plant hosts in numerous countries under various environmental conditions (67). Interestingly, *A. tenuissima*, a fungal endophyte, has been shown to influence growth and selenium absorption in its bioaccumulation host Astragalus bisulcatus (68). The ethyl acetate (EA) crude extract of endogenous A. alternata obtained from Ziziphus spina-christi included alkaloids, tannins, flavonoids, glycosides, phenolics, and terpenoids, according to preliminary phytochemical screening. This demonstrated the potential of *Alternaria* as an important candidate microbial resource for promoting the growth and secondary metabolite accumulation of *P. lactiflora*. Additionally, these can provide the material basis of biological bacterial fertilizer for *P. lactiflora* cultivation. Altogether, further mycobacterial investigations are required to confirm that the bacteria and fungi discovered in this work that are connected to the production of paeoniflorin are indeed candidate microorganisms for enhancing the secondary metabolites of *P. lactiflora*. The study of isolation of *P. lactiflora* endophytes showed that *Alternaria tenuissima*, Aspergillus flavus, and Penicillium commune can all produce paeoniflorin, and A. flavus can produce paeoniflorin in an amount of 342.4 μg/L (69). Furthermore, in this study, BLAST search was used to identify similar sequences of the above three fungi (Fig. S11). In addition, the random forest results indicated that only the genus *Alternaria* was identified in the BS and RS, and it was positively correlated with the content of paeoniflorin.

**FIG 7:**
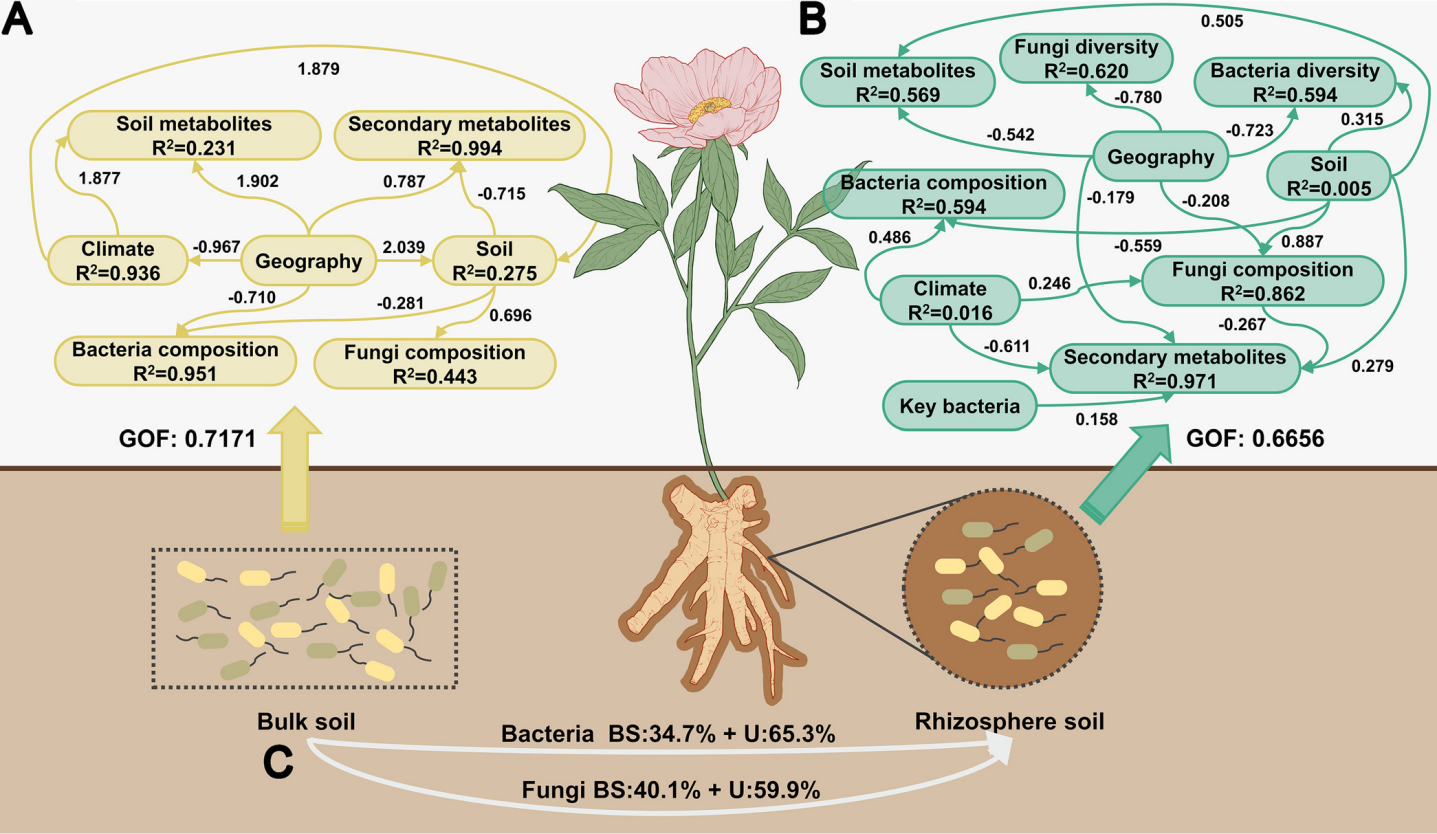
(A and B) Structural equation models (SEMs) illustrate the direct or indirect effects of multiple predictors on *P. lactiflora* root secondary metabolites in the bulk soil (A) and rhizosphere (B). GOF (goodness-of-fit) can be regarded as a measure of the overall explanatory power of the entire model. (C) Source model of plant microbiome (SMPM) showing the potential sources of bacterial and fungal communities from bulk soil to the rhizosphere. U, unknown source.

Two partial least-squares path models (PLS-PMs) were created independently for the bulk soil and rhizosphere to clearly illustrate the regulatory linkages among these factors (Fig. 6A and B). Using the multiple regression analyses outlined in the previous results, a representative for each component in the PLS-PMs was cautiously chosen. Only the variables that were significant were kept in the equations. The goodness-of-fit index (GOF) was used to judge the overall fitting degree of the model, and the results showed that the fitting degrees of the BS (0.7171) and RS (0.6656) were both good. In the BS, geography (path coefficient = 0.787) and soil (−0.715) were seen in a substantial positive and negative major impact on root secondary metabolites (*R*^2^ = 0.994). Soil metabolites (*R*^2^ = 0.231) were directly and positively affected by geography (1.902) and climate (1.8777). Fungal composition (*R*^2^ = 0.443) was affected only by soil (0.696), while bacterial composition (*R*^2^ = 0.951) was influenced by geography (−0.710) and soil (−0.281). The PLS-PM of the RS was more complicated than that of the BS. Root secondary metabolites in the RS model were mostly explained by all factors (*R*^2^ = 0.971) and were significantly affected by the soil (0.279), key bacteria (0.158), climate (−0.611), and geography (−0.179). The direct effects on the soil metabolites (*R*^2^ = 0.569) were climate (−0.542) and soil (0.505). Geography had significant direct influences on bacterial diversity (−0.723) and fungal diversity (−0.780). The soil had the greatest impact on the bacterial composition (−0.559) and fungal composition (0.887). Furthermore, according to the source model of plant microbiome (SMPM) (Fig. 7), bacterial communities in the RS were obtained from the BS (34.7%) and unknown sources (65.3%). The fungal community of the RS contained 40.1% from the BS and 59.9% from unknown sources.

### Conclusion.

In conclusion, this study provides a systematic understanding of the differences and diversity of the bacterial and fungal communities and soil metabolome between the BS and RS at four origins of *P. lactiflora*. The following conclusions were obtained. (i) The abundance of soil metabolism in the RS was different from that in the BS, in which both carbohydrates and organic acids were enriched in the RS. (ii) Copiotrophs were enriched in the rhizosphere, while oligotrophs were enriched in the BS. Similarly, both bacterial and fungal alpha diversity and beta diversity were significantly lower in the RS than in the BS. Additionally, cation exchange capacity (CEC) significantly affected the microbial community diversity of the BS and RS. (iii) Stochastic processes (dispersal limitation) dominated the community assembly of both the RS and BS. (iv) The bacterial-fungal interkingdom network of the RS was more complex and stable than that of the BS. (v) The microbes of high-quality *P. lactiflora* production were deduced by constructing biotic and abiotic model pathways that affect the growth and metabolism of *P. lactiflora*. Meanwhile, multiple abiotic (SWC, MAP, pH, etc.) and biotic (*Alternaria*, etc.) variables influenced the secondary metabolites of *P. lactiflora* roots. Overall, this study strengthens the understanding of the impact of rhizosphere effects on soil composition and diversity. These results can offer an important theoretical basis and technical support for the improvement of *P. lactiflora* quality and future synthetic community studies.

## MATERIALS AND METHODS

### Sampling sites and analyses of environmental characteristics and soil metabolites.

Roots and two root-associated compartments of soil (bulk soil [BS] and rhizosphere [RS]) of *Paeonia lactiflora* were collected from 24 sampling sites in the four major *P. lactiflora* production areas (see Table S12 in the supplemental material) in October 2020. The four chosen origins were Bozhou in Anhui (AH), Zhongjiang in Sichuan (SC), Heze in Shandong (SD), and Pan’an in Zhejiang (ZJ). The sites were collected in a Z pattern, with six (5 by 5 m^2^ per plot) serving as biological duplicates for each planted area (approximately 0.5 ha). The agricultural *P. lactiflora* areas had grown for 4 years with comparable treatment and management measures. Five topsoil cores (0- to 15-cm soil surface depth and ~20 cm away from the roots) and the accompanying intact and healthy roots were collected for each plot. These topsoils from the roots were passed through a sterile 2-mm sieve and were defined as bulk soil (BS). The roots and the soil that could not be shaken off them were put into a 50-mL centrifuge tube. Phosphate-balanced normal saline (PBS) was added to the centrifuge tube. Then, the centrifuge tubes were shaken on the platform (20 min, 180 rpm) (70). After removal of the roots, the remaining samples were centrifuged at 4,000 × *g* for 20 min at 4°C to collect the rhizosphere soil (RS; defined as that 1 mm of soil tightly attached to the roots) (71). For bulk soil (BS), rhizosphere (RS), and root samples, these samples were taken at random and blended as one biological replicate. Overall, 48 samples (four fields × six plots × two compartments) were collected. Before DNA extraction, all samples were collected and transported on dry ice and kept at −80°C. For the investigation of edaphic characteristics and root secondary metabolites, BS and root samples were employed, respectively. The BS and RS samples were used for the soil metabolites and microbial sequencing analyses.

Edaphic physicochemical characteristics (e.g., soil texture, soil water content, pH, and available phosphorus [Table S13]) and soil enzyme activities (e.g., soil catalase and soil urease [Table S13]) of the BS were measured according to previous protocols (72, 73). The meteorological data (Table S13) for all sampling locations, including the mean annual precipitation (MAP), mean annual temperature (MAT), solar radiation (Srad), and wind speed (wind), were taken from the WorldClim database version 2.0 (http://www.worldclim.org) (74).

Soil metabolic composition of the BS and RS was determined by GC-MS using previous methods (75). For the analysis of soil metabolomes of the BS and RS, principal-component analysis (PCA) was performed using the vegan package. Venn diagrams were drawn using the VennDiagram package (76) to identify unique and common soil metabolites. The Bray-Curtis distances of soil metabolites were counted by the vegan package. The EdgeR package (77) was used to calculate the differentially accumulated soil metabolites (DAM) between the BS and RS.

### Analyses of root secondary metabolome.

The major root secondary metabolites (Table S9) were determined and quantification of *P. lactiflora* was performed by liquid chromatography-mass spectrometry (LC-MS). We used 11 standards including albiflorin, benzoyloxypaeoniflorin, benzoylpaeoniflorin, cianidanol, debenzoylpaeoniflorin, ethyl gallate, gallic acid, oxypaeoniflorin, paeoniflorin, paeonilactone A, and paeonol to make standard curves. We used the standard curves to calculate the content in all samples. Chromatographic separations were performed using rapid-separation LC (ThermoFisher Scientific; Dionex UltiMate 3000) with an ultraperformance LC (UPLC) RP column (Waters; Acquity UPLC HSS T3 [1.8 μm, 2.1 mm by 100 mm]) to analyze the samples. Parameters were as follows: mobile phase, A (water, 0.1% formic acid) and B (acetonitrile); elution procedure, 95:5 (vol/vol) at 0 min, 95:5 (vol/vol) at 1 min, 50:50 (vol/vol) at 6 min, 5:95 (vol/vol) at 7 min, 0:100 (vol/vol) at 14 min, 95:5 (vol/vol) at 14.1 min, and 95:5 (vol/vol) at 16 min; flow rate, 0.30 mL min^−1^; injection volume, 3 μL; column temperature, 40°C.

A quadrupole orbital ion trap mass spectrometer coupled with a thermoelectrospray ion source was used for mass spectrometry. The positive ion source voltage (spray voltage) was 3.5 kV, the capillary temperature was 320°C, the negative ion source voltage was 3.0 kV, and the capillary tube heating temperature was 300°C. The sheath gas pressure was 30 lb/in^2^, while the auxiliary gas pressure was 10 lb/in^2^. The temperature of volume heating evaporation was 300°C. Nitrogen is present in both the air and the auxiliary gas. At a pressure of 1.5 mtorr, the collision gas was nitrogen. The first-level full scan parameters were as follows: resolution, 70,000; automatic gain control target, 1 × 10^6^; maximum isolation time, 50 ms; and mass-to-charge ratio scan range, 80 to 1,200 Da. The LC-MS system was managed by the Xcalibur 2.2 SP1.48 software, which also regulated data gathering.

### Bacterial and fungal amplicon sequencing and statistical analysis.

HiPure soil DNA kits (Magen, Guangzhou, China) were used to extract total DNA from soil samples (0.5 g each) for the BS and RS. The primers for amplifying bacteria were 341F (CCTACGGGNGGCWGCAG) and 806R (GGACTACHVGGGTATCTAAT) in the V3-V4 hypervariable region of 16S rRNA (78), while the primers for amplifying fungi were ITS3_KYO2 (GGAAGTAAAAGTCGTAACAAGG) and TS4 (TCCTCCGCTTATTGATATGC) in the ITS2 region (79). The paired-end approach was used for sequencing using the Illumina Novaseq 6000 sequencing platform. The raw reads were uploaded to the NCBI Sequence Read Archive (SRA) database (accession number PRJNA855546).

The pipeline of the DADA2 R package (80) was used to quality control, assemble, and merge the sequencing data into the original amplicon sequence variants (ASVs) with a minimum overlap of 12 bp. The denoised, chimera-free ASV sequences and their abundances were produced following chimera removal by the UCHIME method (80). Annotated classification of bacteria and fungi was performed using the SILVA database (81) (version 13.2) and the UNITE database (82) (version 8.0). Further, only the sequences annotated to bacterial and fungal domains were retained, which were 130,053 and 7,559, respectively (supplemental material). ASV tables for bacteria and fungi were diluted to 52,072 and 49,947 reads by using the vegan (83) package (version 2.6-2). The Chao1 index was calculated for assessing alpha diversity. The cumulative and scaling (CSS) function of metagenomeSeq was used as a normalization method for ASVs (84).

All statistical analyses were carried out in the R environment (v4.1.3; http://www.r-project.org/). The ggplot2 package was used to illustrate the majority of the findings. Both ANOVA and posterior analysis calculations were performed using the EasyStat package (https://github.com/taowenmicro/EasyStat). Furthermore, the false-discovery rate approach was used to correct all of the *P* values (85). The classification tree of ASVs was drawn with Cytoscape (77). The Chao1 index, computed with the picante package, was used to examine the richness of species diversity (alpha diversity) (86). The equilibrium of the microbial communities was assessed using the bacterium/fungus ratio of the Chao1 index. Latitude patterns of alpha diversity of microbial communities were analyzed using the vegan package and linear regression. Correlations between alpha diversity and environmental variables, as well as bacterial and fungal alpha diversity, were analyzed using Spearman’s coefficient and linear regression. The beta diversity of microbes was calculated by computing the Bray-Curtis distance matrix and then reconciled using adjusted principal-coordinate analysis (aPCoA) (44), which excluded the effect of sampling location. Both analysis of molecular variance (AMOVA) and PERMANOVA were performed by the vegan package with 999 permutations. The relationship between bacterial and fungal community composition (Bray-Curtis dissimilarity) was explored using both the Mantel test and linear regression. Bacterial and fungal data were examined for association using the Procrustes analysis of the vegan package. The univariate linear regression analysis was used to study the distance-decay relationships (DDRs) between microbial distribution (Bray-Curtis distance) similarity and geographical location, where the slope of the DDR might change from compartment to compartment, representing various species’ turnover rates. Compositional differences between compartments were divided into replacement and richness difference components (Podani family, Sørensen dissimilarities) by the ade4 package (87, 88).

The assembly process of bacterial and fungal communities was calculated using a null model (89). According to βNTI and RC_Bray_ (Bray-Curtis), the ecological process was divided into five processes: stochastic processes (dispersal limitation, homogenizing dispersal, and undominated) and deterministic processes (heterogeneous selection and homogeneous selection) (10). To assess whether dispersal or niche processes dominated community construction, calculations were performed using the DNCI package (90). The modified stochastic ratio (MST), which was used for the relative importance of deterministic (MST < 0.5) and stochastic (MST > 0.5) processes, was computed by the NST package (91). The neutral community model (NCM), niche breadth calculations, and niche generalist and specialist divisions were performed according to our previous study (92).

The analyses of the bacterial-fungal interkingdom networks of the BS and RS were conducted by using the interdomain ecological network analysis pipeline (IDENAP) (93) on iNAP (https://onlinelibrary.wiley.com/doi/full/10.1002/imt2.13). The interkingdom networks were constructed using the correlation coefficient *r* of >0.6 as the threshold based on the SparCC method. In addition, the bipartite networks were also constructed based on the above-described method. According to previous methods (92), the nodes in the network were classified according to within-module connectivity (*Z_i_*) and among-module connectivity (*P_i_*): module hubs, peripherals, connectors, and network hubs. The relationship (the number of edges) between bacteria and bacteria, fungi and fungi, and bacteria and fungi was calculated according to previous studies (94). The complexity of the interkingdom network (e.g., total number of nodes, total number of links, and average connectivity [Table S3]) was calculated by the igraph package (95). Natural connectivity was used to compare the robustness values of the interkingdom network (96). Furthermore, the complexity after randomly removing 50% of nodes and after targeted removal of module hubs and vulnerability were also calculated according to the previous study (24). The cohesion was calculated based on the null model (97).

Using the linkET package, the Mantel test was used to investigate Spearman’s linkages between root secondary metabolites and environmental variables (microbes, soil metabolites, etc.). Multiple reaction monitoring (MRM) analysis was utilized to explore the influence of substantially correlated factors on microbes (Bray-Curtis distance) within every compartment by the ecodist package (98). Canonical correspondence analysis (CCA) was used to investigate the relative impact of significant environmental variables on microbial composition. The relative importance of explanatory variables for canonical analysis was obtained using the hierarchical partitioning of the rdacca.hp package, and the UpSetVP package was used to visualize the results (99). For the selected important environmental variables, PCoA and smooth regression analysis of additive models were performed using previous methods.

For the macroenvironmental data, the selection was based on the correlation and random forest model results with the secondary metabolites of *P. lactiflora* root. Random forest regression was performed through rfPermute and A3 packages to obtain the significance of variables and the full model, respectively. The mean annual precipitation (MAP) map of sampling sites was drawn with ArcMap 10.7 (100). The univariate regression on secondary metabolites of *P. lactiflora* root and environmental variables was performed with the ‘lm()’ function of R. To obtain relevant genera of paeoniflorin-related fungi and bacteria in the BS and RS, the RandomForest software was employed, and fitted curves were utilized to explain the link between the abundance of the 10 leading genera and paeoniflorin concentration. Key modules significantly associated with root secondary metabolites were obtained using WGCNA (101). ASV and root secondary metabolites in the above key modules were analyzed by the random forest model through the linkET package. The cooccurrence network based on the key modules was constructed through the igraph package. BLAST searching on NCBI (https://blast.ncbi.nlm.nih.gov/Blast.cgi) properly identified the important ASVs, and the maximum likelihood trees (ML trees) were generated by using sequences with the greatest recognition scores. Ridgeline plots show the relative abundance of key ASVs. Paeoniflorin-related fungi reported in the previous study (69) were screened from fungal sequences by local BLAST search, and maximum likelihood trees were constructed. Finally, the plspm tool was used to create partial least-squares path models to recapitulate the link among root secondary metabolites, soil metabolites, microbiome, and environment (102). The goodness-of-fit index (GOF) was generated to assess the model’s overall fit.

### Data availability.

The raw sequence data reported in this paper are available in the NCBI Sequence Read Archive under accession no. PRJNA855546.

## References

[1] Ling N, Wang T, Kuzyakov Y. 2022. Rhizosphere bacteriome structure and functions. Nat Commun 13:836. doi:10.1038/s41467-022-28448-9. 35149704 PMC8837802

[2] Howard MM, Muñoz CA, Kao-Kniffin J, Kessler A. 2020. Soil microbiomes from fallow fields have species-specific effects on crop growth and pest resistance. Front Plant Sci 11:1171. doi:10.3389/fpls.2020.01171. 32849726 PMC7419683

[3] Peiffer JA, Spor A, Koren O, Jin Z, Tringe SG, Dangl JL, Buckler ES, Ley RE. 2013. Diversity and heritability of the maize rhizosphere microbiome under field conditions. Proc Natl Acad Sci USA 110:6548–6553. doi:10.1073/pnas.1302837110. 23576752 PMC3631645

[4] Vieira S, Sikorski J, Dietz S, Herz K, Schrumpf M, Bruelheide H, Scheel D, Friedrich MW, Overmann J. 2020. Drivers of the composition of active rhizosphere bacterial communities in temperate grasslands. ISME J 14:463–475. doi:10.1038/s41396-019-0543-4. 31659233 PMC6976627

[5] Bais HP, Weir TL, Perry LG, Gilroy S, Vivanco JM. 2006. The role of root exudates in rhizosphere interactions with plants and other organisms. Annu Rev Plant Biol 57:233–266. doi:10.1146/annurev.arplant.57.032905.105159. 16669762

[6] Yan Y, Kuramae EE, de Hollander M, Klinkhamer PG, van Veen JA. 2017. Functional traits dominate the diversity-related selection of bacterial communities in the rhizosphere. ISME J 11:56–66. doi:10.1038/ismej.2016.108. 27482928 PMC5315473

[7] Fan K, Weisenhorn P, Gilbert JA, Chu H. 2018. Wheat rhizosphere harbors a less complex and more stable microbial co-occurrence pattern than bulk soil. Soil Biol Biochem 125:251–260. doi:10.1016/j.soilbio.2018.07.022.

[8] Ren Y, Xun W, Yan H, Ma A, Xiong W, Shen Q, Zhang R. 2020. Functional compensation dominates the assembly of plant rhizospheric bacterial community. Soil Biol Biochem 150:107968. doi:10.1016/j.soilbio.2020.107968.

[9] Vorholt JA, Vogel C, Carlström CI, Müller DB. 2017. Establishing causality: opportunities of synthetic communities for plant microbiome research. Cell Host Microbe 22:142–155. doi:10.1016/j.chom.2017.07.004. 28799900

[10] Xiong C, Singh BK, He J-Z, Han Y-L, Li P-P, Wan L-H, Meng G-Z, Liu S-Y, Wang J-T, Wu C-F, Ge A-H, Zhang L-M. 2021. Plant developmental stage drives the differentiation in ecological role of the maize microbiome. Microbiome 9:171. doi:10.1186/s40168-021-01118-6. 34389047 PMC8364065

[11] Chen H, Wu H, Yan B, Zhao H, Liu F, Zhang H, Sheng Q, Miao F, Liang Z. 2018. Core microbiome of medicinal plant Salvia miltiorrhiza seed: a rich reservoir of beneficial microbes for secondary metabolism? Int J Mol Sci 19:672. doi:10.3390/ijms19030672. 29495531 PMC5877533

[12] Xie W, Hao Z, Zhou X, Jiang X, Xu L, Wu S, Zhao A, Zhang X, Chen B. 2018. Arbuscular mycorrhiza facilitates the accumulation of glycyrrhizin and liquiritin in Glycyrrhiza uralensis under drought stress. Mycorrhiza 28:285–300. doi:10.1007/s00572-018-0827-y. 29455337

[13] Xu J, Zhang Y, Zhang P, Trivedi P, Riera N, Wang Y, Liu X, Fan G, Tang J, Coletta-Filho HD, Cubero J, Deng X, Ancona V, Lu Z, Zhong B, Roper MC, Capote N, Catara V, Pietersen G, Vernière C, Al-Sadi AM, Li L, Yang F, Xu X, Wang J, Yang H, Jin T, Wang N. 2018. The structure and function of the global citrus rhizosphere microbiome. Nat Commun 9:4894. doi:10.1038/s41467-018-07343-2. 30459421 PMC6244077

[14] Zhang G, Wei F, Chen Z, Wang Y, Jiao S, Yang J, Chen Y, Liu C, Huang Z, Dong L, Chen S. 2022. Evidence for saponin diversity-mycobiome links and conservatism of plant-fungi interaction patterns across Holarctic disjunct Panax species. Sci Total Environ 830:154583. doi:10.1016/j.scitotenv.2022.154583. 35304141

[15] Liu Y, Li D, Gao H, Li Y, Chen W, Jiao S, Wei G. 2022. Regulation of soil micro-foodwebs to root secondary metabolites in cultivated and wild licorice plants. Sci Total Environ 828:154302. doi:10.1016/j.scitotenv.2022.154302. 35276159

[16] Chandel A, Thakur M, Singh G, Dogra R, Bajad A, Soni V, Bhargava B. 2022. Flower regulation in floriculture: an agronomic concept and commercial use. J Plant Growth Regul doi:10.1007/s00344-022-10688-0.

[17] Kamenetsky R, Dole J. 2012. Herbaceous peony (Paeonia): genetics, physiology and cut flower production. Floric Ornam Biotechnol 6:62–77.

[18] He D-Y, Dai S-M. 2011. Anti-inflammatory and immunomodulatory effects of Paeonia lactiflora Pall., a traditional Chinese herbal medicine. Front Pharmacol 2:10. doi:10.3389/fphar.2011.00010. 21687505 PMC3108611

[19] Liu B, Meng X, Ma Y, Li H, Liu Y, Shi N, Chen Y, Wang Y, Lu C. 2021. Clinical safety of total glucosides of paeony adjuvant therapy for rheumatoid arthritis treatment: a systematic review and meta-analysis. BMC Complement Med Ther 21:102. doi:10.1186/s12906-021-03252-y. 33771151 PMC8004450

[20] Liu K, Ding X, Wang J. 2020. Soil metabolome correlates with bacterial diversity and co-occurrence patterns in root-associated soils on the Tibetan Plateau. Sci Total Environ 735:139572. doi:10.1016/j.scitotenv.2020.139572. 32480142

[21] Kuzyakov Y, Razavi BS. 2019. Rhizosphere size and shape: temporal dynamics and spatial stationarity. Soil Biol Biochem 135:343–360. doi:10.1016/j.soilbio.2019.05.011.

[22] Baran R, Brodie EL, Mayberry-Lewis J, Hummel E, Da Rocha UN, Chakraborty R, Bowen BP, Karaoz U, Cadillo-Quiroz H, Garcia-Pichel F, Northen TR. 2015. Exometabolite niche partitioning among sympatric soil bacteria. Nat Commun 6:8289. doi:10.1038/ncomms9289. 26392107 PMC4595634

[23] Withers E, Hill PW, Chadwick DR, Jones DL. 2020. Use of untargeted metabolomics for assessing soil quality and microbial function. Soil Biol Biochem 143:107758. doi:10.1016/j.soilbio.2020.107758.

[24] Yuan MM, Guo X, Wu L, Zhang Y, Xiao N, Ning D, Shi Z, Zhou X, Wu L, Yang Y, Tiedje JM, Zhou J. 2021. Climate warming enhances microbial network complexity and stability. Nat Clim Chang 11:343–348. doi:10.1038/s41558-021-00989-9.

[25] Dennis PG, Miller AJ, Hirsch PR. 2010. Are root exudates more important than other sources of rhizodeposits in structuring rhizosphere bacterial communities? FEMS Microbiol Ecol 72:313–327. doi:10.1111/j.1574-6941.2010.00860.x. 20370828

[26] Bakker PA, Berendsen RL, Doornbos RF, Wintermans PC, Pieterse CM. 2013. The rhizosphere revisited: root microbiomics. Front Plant Sci 4:165. doi:10.3389/fpls.2013.00165. 23755059 PMC3667247

[27] Carvalhais LC, Dennis PG, Fedoseyenko D, Hajirezaei MR, Borriss R, von Wirén N. 2011. Root exudation of sugars, amino acids, and organic acids by maize as affected by nitrogen, phosphorus, potassium, and iron deficiency. J Plant Nutr Soil Sci 174:3–11. doi:10.1002/jpln.201000085.

[28] Gouffi K, Bernard T, Blanco C. 2000. Osmoprotection by pipecolic acid in Sinorhizobium meliloti: specific effects of d and l isomers. Appl Environ Microbiol 66:2358–2364. doi:10.1128/AEM.66.6.2358-2364.2000. 10831411 PMC110532

[29] Ruan Y-L. 2014. Sucrose metabolism: gateway to diverse carbon use and sugar signaling. Annu Rev Plant Biol 65:33–67. doi:10.1146/annurev-arplant-050213-040251. 24579990

[30] Shi S, Richardson AE, O’Callaghan M, DeAngelis KM, Jones EE, Stewart A, Firestone MK, Condron LM. 2011. Effects of selected root exudate components on soil bacterial communities. FEMS Microbiol Ecol 77:600–610. doi:10.1111/j.1574-6941.2011.01150.x. 21658090

[31] Jin Y, Zhu H, Luo S, Yang W, Zhang L, Li S, Jin Q, Cao Q, Sun S, Xiao M. 2019. Role of maize root exudates in promotion of colonization of Bacillus velezensis strain S3-1 in rhizosphere soil and root tissue. Curr Microbiol 76:855–862. doi:10.1007/s00284-019-01699-4. 31073734

[32] Jacoby RP, Kopriva S. 2019. Metabolic niches in the rhizosphere microbiome: new tools and approaches to analyse metabolic mechanisms of plant–microbe nutrient exchange. J Exp Bot 70:1087–1094. doi:10.1093/jxb/ery438. 30576534

[33] Macabuhay A, Arsova B, Walker R, Johnson A, Watt M, Roessner U. 2022. Modulators or facilitators? Roles of lipids in plant root-microbe interactions. Trends Plant Sci 27:180–190. doi:10.1016/j.tplants.2021.08.004. 34620547

[34] van Dam NM, Bouwmeester HJ. 2016. Metabolomics in the rhizosphere: tapping into belowground chemical communication. Trends Plant Sci 21:256–265. doi:10.1016/j.tplants.2016.01.008. 26832948

[35] He H, Zhu W, Noor I, Liu J, Li G. 2019. Pseudomonas putida WH-B3 degrades benzoic acid and alleviates its autotoxicity to peach (Prunus persica L. batsch) seedlings grown in replanted soil. Sci Hortic 255:183–192. doi:10.1016/j.scienta.2019.05.020.

[36] Song Y, Li X, Yao S, Yang X, Jiang X. 2020. Correlations between soil metabolomics and bacterial community structures in the pepper rhizosphere under plastic greenhouse cultivation. Sci Total Environ 728:138439. doi:10.1016/j.scitotenv.2020.138439. 32361108

[37] Chen Y, Neilson JW, Kushwaha P, Maier RM, Barberán A. 2021. Life-history strategies of soil microbial communities in an arid ecosystem. ISME J 15:649–657. doi:10.1038/s41396-020-00803-y. 33051582 PMC8027408

[38] Koch AL. 2001. Oligotrophs versus copiotrophs. Bioessays 23:657–661. doi:10.1002/bies.1091. 11462219

[39] Ho A, Di Lonardo DP, Bodelier PL. 2017. Revisiting life strategy concepts in environmental microbial ecology. FEMS Microbiol Ecol 93:fix006. doi:10.1093/femsec/fix006. 28115400

[40] Egidi E, Delgado-Baquerizo M, Plett JM, Wang J, Eldridge DJ, Bardgett RD, Maestre FT, Singh BK. 2019. A few Ascomycota taxa dominate soil fungal communities worldwide. Nat Commun 10:2369. doi:10.1038/s41467-019-10373-z. 31147554 PMC6542806

[41] Schoch CL, Sung G-H, López-Giráldez F, Townsend JP, Miadlikowska J, Hofstetter V, Robbertse B, Matheny PB, Kauff F, Wang Z, Gueidan C, Andrie RM, Trippe K, Ciufetti LM, Wynns A, Fraker E, Hodkinson BP, Bonito G, Groenewald JZ, Arzanlou M, de Hoog GS, Crous PW, Hewitt D, Pfister DH, Peterson K, Gryzenhout M, Wingfield MJ, Aptroot A, Suh S-O, Blackwell M, Hillis DM, Griffith GW, Castlebury LA, Rossman AY, Lumbsch HT, Lücking R, Büdel B, Rauhut A, Diederich P, Ertz D, Geiser DM, Hosaka K, Inderbitzin P, Kohlmeyer J, Volkmann-Kohlmeyer B, Mostert L, O'Donnell K, Sipman H, Rogers JD, Shoemaker RA, Sugiyama J, Summerbell RC, Untereiner W, Johnston PR, Stenroos S, Zuccaro A, Dyer PS, Crittenden PD, Cole MS, Hansen K, Trappe JM, Yahr R, Lutzoni F, Spatafora JW. 2009. The Ascomycota tree of life: a phylum-wide phylogeny clarifies the origin and evolution of fundamental reproductive and ecological traits. Syst Biol 58:224–239. doi:10.1093/sysbio/syp020. 20525580

[42] Applebaum I, Jeyaraman M, Sherman C, Doniger T, Steinberger Y. 2022. Structure and function of the soil rhizosphere fungal communities in medicinal plants—a preliminary study. Agriculture 12:152. doi:10.3390/agriculture12020152.

[43] Nautiyal CS, Dion P (ed). 2008. Molecular mechanisms of plant and microbe coexistence. Soil biology, vol 15. Springer, Berlin, Germany.

[44] Shi Y, Zhang L, Do K-A, Peterson CB, Jenq RR. 2020. aPCoA: covariate adjusted principal coordinates analysis. Bioinformatics 36:4099–4101. doi:10.1093/bioinformatics/btaa276. 32339223 PMC7332564

[45] Zhang J, Zhang B, Liu Y, Guo Y, Shi P, Wei G. 2018. Distinct large-scale biogeographic patterns of fungal communities in bulk soil and soybean rhizosphere in China. Sci Total Environ 644:791–800. doi:10.1016/j.scitotenv.2018.07.016. 29990927

[46] Xun W, Shao J, Shen Q, Zhang R. 2021. Rhizosphere microbiome: functional compensatory assembly for plant fitness. Comput Struct Biotechnol J 19:5487–5493. doi:10.1016/j.csbj.2021.09.035. 34712394 PMC8515068

[47] Legendre P. 2014. Interpreting the replacement and richness difference components of beta diversity. Glob Ecol Biogeogr 23:1324–1334. doi:10.1111/geb.12207.

[48] Brunel C, Pouteau R, Dawson W, Pester M, Ramirez KS, van Kleunen M. 2020. Towards unraveling macroecological patterns in rhizosphere microbiomes. Trends Plant Sci 25:1017–1029. doi:10.1016/j.tplants.2020.04.015. 32467065

[49] Hubbell SP. 2011. The unified neutral theory of biodiversity and biogeography (MPB-32). Princeton University Press, Princeton, NJ.

[50] Baldeck CA, Harms KE, Yavitt JB, John R, Turner BL, Valencia R, Navarrete H, Davies SJ, Chuyong GB, Kenfack D, Thomas DW, Madawala S, Gunatilleke N, Gunatilleke S, Bunyavejchewin S, Kiratiprayoon S, Yaacob A, Supardi MNN, Dalling JW. 2013. Soil resources and topography shape local tree community structure in tropical forests. Proc Biol Sci 280:20122532. doi:10.1098/rspb.2012.2532. 23256196 PMC3574348

[51] Zhou J, Ning D. 2017. Stochastic community assembly: does it matter in microbial ecology? Microbiol Mol Biol Rev 81:e00002-17. doi:10.1128/MMBR.00002-17. 29021219 PMC5706748

[52] Chase JM, Myers JA. 2011. Disentangling the importance of ecological niches from stochastic processes across scales. Philos Trans R Soc Lond B Biol Sci 366:2351–2363. doi:10.1098/rstb.2011.0063. 21768151 PMC3130433

[53] Hu D, Jiang L, Hou Z, Zhang J, Wang H, Lv G. 2022. Environmental filtration and dispersal limitation explain different aspects of beta diversity in desert plant communities. Glob Ecol Conserv 33:e01956. doi:10.1016/j.gecco.2021.e01956.

[54] Thiergart T, Durán P, Ellis T, Vannier N, Garrido-Oter R, Kemen E, Roux F, Alonso-Blanco C, Ågren J, Schulze-Lefert P, Hacquard S. 2020. Root microbiota assembly and adaptive differentiation among European Arabidopsis populations. Nat Ecol Evol 4:122–131. doi:10.1038/s41559-019-1063-3. 31900452

[55] Chen Q-L, Hu H-W, Yan Z-Z, Li C-Y, Nguyen B-AT, Sun A-Q, Zhu Y-G, He J-Z. 2021. Deterministic selection dominates microbial community assembly in termite mounds. Soil Biol Biochem 152:108073. doi:10.1016/j.soilbio.2020.108073.

[56] Farjalla VF, Srivastava DS, Marino NA, Azevedo FD, Dib V, Lopes PM, Rosado AS, Bozelli RL, Esteves FA. 2012. Ecological determinism increases with organism size. Ecology 93:1752–1759. doi:10.1890/11-1144.1. 22919920

[57] Mo Y, Peng F, Gao X, Xiao P, Logares R, Jeppesen E, Ren K, Xue Y, Yang J. 2021. Low shifts in salinity determined assembly processes and network stability of microeukaryotic plankton communities in a subtropical urban reservoir. Microbiome 9:128. doi:10.1186/s40168-021-01079-w. 34082826 PMC8176698

[58] Martiny JB, Eisen JA, Penn K, Allison SD, Horner-Devine MC. 2011. Drivers of bacterial β-diversity depend on spatial scale. Proc Natl Acad Sci USA 108:7850–7854. doi:10.1073/pnas.1016308108. 21518859 PMC3093525

[59] Feng M, Tripathi BM, Shi Y, Adams JM, Zhu YG, Chu H. 2019. Interpreting distance-decay pattern of soil bacteria via quantifying the assembly processes at multiple spatial scales. Microbiologyopen 8:e00851. doi:10.1002/mbo3.851. 31074596 PMC6741136

[60] Hanson CA, Fuhrman JA, Horner-Devine MC, Martiny JB. 2012. Beyond biogeographic patterns: processes shaping the microbial landscape. Nat Rev Microbiol 10:497–506. doi:10.1038/nrmicro2795. 22580365

[61] Zarraonaindia I, Owens SM, Weisenhorn P, West K, Hampton-Marcell J, Lax S, Bokulich NA, Mills DA, Martin G, Taghavi S, van der Lelie D, Gilbert JA. 2015. The soil microbiome influences grapevine-associated microbiota. mBio 6:e02527-14. doi:10.1128/mBio.02527-14. 25805735 PMC4453523

[62] Bai B, Liu W, Qiu X, Zhang J, Zhang J, Bai Y. 2022. The root microbiome: community assembly and its contributions to plant fitness. J Integr Plant Biol 64:230–243. doi:10.1111/jipb.13226. 35029016

[63] Zhang G, Wei G, Wei F, Chen Z, He M, Jiao S, Wang Y, Dong L, Chen S. 2021. Dispersal limitation plays stronger role in the community assembly of fungi relative to bacteria in rhizosphere across the arable area of medicinal plant. Front Microbiol 12:713523. doi:10.3389/fmicb.2021.713523. 34484152 PMC8415459

[64] Li Y, Kong D, Fu Y, Sussman MR, Wu H. 2020. The effect of developmental and environmental factors on secondary metabolites in medicinal plants. Plant Physiol Biochem 148:80–89. doi:10.1016/j.plaphy.2020.01.006. 31951944

[65] Verma N, Shukla S. 2015. Impact of various factors responsible for fluctuation in plant secondary metabolites. J Appl Res Med Aromat Plants 2:105–113. doi:10.1016/j.jarmap.2015.09.002.

[66] Pang Z, Chen J, Wang T, Gao C, Li Z, Guo L, Xu J, Cheng Y. 2021. Linking plant secondary metabolites and plant microbiomes: a review. Front Plant Sci 12:621276. doi:10.3389/fpls.2021.621276. 33737943 PMC7961088

[67] Chełkowski J, Visconti A. 1992. Alternaria: biology, plant diseases, and metabolites, vol 3. Elsevier Science Limited, Amsterdam, Netherlands.

[68] Lindblom SD, Wangeline AL, Valdez Barillas JR, Devilbiss B, Fakra SC, Pilon-Smits EA. 2018. Fungal endophyte Alternaria tenuissima can affect growth and selenium accumulation in its hyperaccumulator host Astragalus bisulcatus. Front Plant Sci 9:1213. doi:10.3389/fpls.2018.01213. 30177943 PMC6109757

[69] Cheng X, Wei Z, Pu S, Xiang M, Yan A, Zhang Y, Wang X. 2018. Diversity of endophytic fungi of Paeonia lactiflora Pallas and screening for fungal paeoniflorin producers. FEMS Microbiol Lett 365:fny263. doi:10.1093/femsle/fny263. 30445580

[70] Beckers B, Op De Beeck M, Weyens N, Boerjan W, Vangronsveld J. 2017. Structural variability and niche differentiation in the rhizosphere and endosphere bacterial microbiome of field-grown poplar trees. Microbiome 5:25. doi:10.1186/s40168-017-0241-2. 28231859 PMC5324219

[71] Edwards J, Johnson C, Santos-Medellín C, Lurie E, Podishetty NK, Bhatnagar S, Eisen JA, Sundaresan V. 2015. Structure, variation, and assembly of the root-associated microbiomes of rice. Proc Natl Acad Sci USA 112:E911–E920. doi:10.1073/pnas.1414592112. 25605935 PMC4345613

[72] Bottomley PJ, Angle JS, Weaver R (ed). 2020. Methods of soil analysis, part 2: microbiological and biochemical properties, vol 12. John Wiley & Sons, Hoboken, NJ.

[73] Liu Y, Zhang L, Lu J, Chen W, Wei G, Lin Y. 2020. Topography affects the soil conditions and bacterial communities along a restoration gradient on Loess-Plateau. Appl Soil Ecol 150:103471. doi:10.1016/j.apsoil.2019.103471.

[74] Fick SE, Hijmans RJ. 2017. WorldClim 2: new 1-km spatial resolution climate surfaces for global land areas. Int J Climatol 37:4302–4315. doi:10.1002/joc.5086.

[75] Shi R, Gu H, He S, Xiong B, Huang Y, Horowitz AR, He X. 2021. Comparative metagenomic and metabolomic profiling of rhizospheres of Panax notoginseng grown under forest and field conditions. Agronomy 11:2488. doi:10.3390/agronomy11122488.

[76] Chen H, Boutros PC. 2011. VennDiagram: a package for the generation of highly-customizable Venn and Euler diagrams in R. BMC Bioinformatics 12:35. doi:10.1186/1471-2105-12-35. 21269502 PMC3041657

[77] Shannon P, Markiel A, Ozier O, Baliga NS, Wang JT, Ramage D, Amin N, Schwikowski B, Ideker T. 2003. Cytoscape: a software environment for integrated models of biomolecular interaction networks. Genome Res 13:2498–2504. doi:10.1101/gr.1239303. 14597658 PMC403769

[78] Guo M, Wu F, Hao G, Qi Q, Li R, Li N, Wei L, Chai T. 2017. Bacillus subtilis improves immunity and disease resistance in rabbits. Front Immunol 8:354. doi:10.3389/fimmu.2017.00354. 28424690 PMC5372816

[79] Toju H, Tanabe AS, Yamamoto S, Sato H. 2012. High-coverage ITS primers for the DNA-based identification of ascomycetes and basidiomycetes in environmental samples. PLoS One 7:e40863. doi:10.1371/journal.pone.0040863. 22808280 PMC3395698

[80] Callahan BJ, McMurdie PJ, Rosen MJ, Han AW, Johnson AJA, Holmes SP. 2016. DADA2: high-resolution sample inference from Illumina amplicon data. Nat Methods 13:581–583. doi:10.1038/nmeth.3869. 27214047 PMC4927377

[81] Pruesse E, Quast C, Knittel K, Fuchs BM, Ludwig W, Peplies J, Glöckner FO. 2007. SILVA: a comprehensive online resource for quality checked and aligned ribosomal RNA sequence data compatible with ARB. Nucleic Acids Res 35:7188–7196. doi:10.1093/nar/gkm864. 17947321 PMC2175337

[82] Edgar RC, Haas BJ, Clemente JC, Quince C, Knight R. 2011. UCHIME improves sensitivity and speed of chimera detection. Bioinformatics 27:2194–2200. doi:10.1093/bioinformatics/btr381. 21700674 PMC3150044

[83] Oksanen J, Blanchet FG, Kindt R, Legendre P, Minchin PR, O’hara RB, Oksanen MJ. 2013. Package ‘vegan’. Community ecology package, version, 2:1–295.

[84] Paulson JN, Pop M, Bravo HC. 2013. metagenomeSeq: statistical analysis for sparse high-throughput sequencing. Bioconductor package.

[85] Benjamini Y, Hochberg Y. 1995. Controlling the false discovery rate: a practical and powerful approach to multiple testing. J R Stat Soc Ser B (Methodol) 57:289–300. doi:10.1111/j.2517-6161.1995.tb02031.x.

[86] Kembel SW, Cowan PD, Helmus MR, Cornwell WK, Morlon H, Ackerly DD, Blomberg SP, Webb CO. 2010. Picante: R tools for integrating phylogenies and ecology. Bioinformatics 26:1463–1464. doi:10.1093/bioinformatics/btq166. 20395285

[87] Shen C, Gunina A, Luo Y, Wang J, He JZ, Kuzyakov Y, Hemp A, Classen AT, Ge Y. 2020. Contrasting patterns and drivers of soil bacterial and fungal diversity across a mountain gradient. Environ Microbiol 22:3287–3301. doi:10.1111/1462-2920.15090. 32436332

[88] Dray S, Dufour A-B. 2007. The ade4 package: implementing the duality diagram for ecologists. J Stat Softw 22:1–20. doi:10.18637/jss.v022.i04.

[89] Stegen JC, Lin X, Fredrickson JK, Chen X, Kennedy DW, Murray CJ, Rockhold ML, Konopka A. 2013. Quantifying community assembly processes and identifying features that impose them. ISME J 7:2069–2079. doi:10.1038/ismej.2013.93. 23739053 PMC3806266

[90] Vilmi A, Gibert C, Escarguel G, Happonen K, Heino J, Jamoneau A, Passy SI, Picazo F, Soininen J, Tison-Rosebery J, Wang J. 2021. Dispersal–niche continuum index: a new quantitative metric for assessing the relative importance of dispersal versus niche processes in community assembly. Ecography 44:370–379. doi:10.1111/ecog.05356.

[91] Ning D, Deng Y, Tiedje JM, Zhou J. 2019. A general framework for quantitatively assessing ecological stochasticity. Proc Natl Acad Sci USA 116:16892–16898. doi:10.1073/pnas.1904623116. 31391302 PMC6708315

[92] Sun X, Pei J, Zhao L, Ahmad B, Huang LF. 2021. Fighting climate change: soil bacteria communities and topography play a role in plant colonization of desert areas. Environ Microbiol 23:6876–6894. doi:10.1111/1462-2920.15799. 34693620

[93] Feng K, Peng X, Zhang Z, Gu S, He Q, Shen W, Wang Z, Wang D, Hu Q, Li Y, Wang S, Deng Y. 2022. iNAP: an integrated network analysis pipeline for microbiome studies. iMeta 1:e13. doi:10.1002/imt2.13. PMC10989900 38868563

[94] Kim H, Lee KK, Jeon J, Harris WA, Lee Y-H. 2020. Domestication of Oryza species eco-evolutionarily shapes bacterial and fungal communities in rice seed. Microbiome 8:20. doi:10.1186/s40168-020-00805-0. 32059747 PMC7023700

[95] Csardi G, Nepusz T. 2006. The igraph software package for complex network research. InterJournal Complex Systems 1695:1–9.

[96] Wu J, Barahona M, Tan Y, Deng H. 2010. Robustness of random graphs based on natural connectivity. doi:10.48550/arXiv.1009.3430. 23278036

[97] Hernandez DJ, David AS, Menges ES, Searcy CA, Afkhami ME. 2021. Environmental stress destabilizes microbial networks. ISME J 15:1722–1734. doi:10.1038/s41396-020-00882-x. 33452480 PMC8163744

[98] Goslee SC, Urban DL. 2007. The ecodist package for dissimilarity-based analysis of ecological data. J Stat Softw 22:1–19. doi:10.18637/jss.v022.i07.

[99] Lai J, Zou Y, Zhang J, Peres-Neto PR. 2022. Generalizing hierarchical and variation partitioning in multiple regression and canonical analyses using the rdacca. hp R package. Methods Ecol Evol 13:782–788. doi:10.1111/2041-210X.13800.

[100] Scott LM, Janikas MV. 2010. Spatial statistics in ArcGIS, p 27–41. *In* Handbook of applied spatial analysis. Springer, Berlin, Germany.

[101] Langfelder P, Horvath S. 2008. WGCNA: an R package for weighted correlation network analysis. BMC Bioinformatics 9:559. doi:10.1186/1471-2105-9-559. 19114008 PMC2631488

[102] Sanchez G, Trinchera L, Sanchez MG, FactoMineR S. 2013. Package ‘plspm’. Citeseer, State College, PA.

