# Supplementary material for: Environmental Response to Root Secondary Metabolite Accumulation in Paeonia lactiflora: Insights from Rhizosphere Metabolism and Root-Associated Microbial Communities
Source: Microbiol Spectr. 2022 Nov 1;10(6):e02800-22. doi: 10.1128/spectrum.02800-22 (PMC9769548; doi:10.1128/spectrum.02800-22)
Supplement: Supplemental file 1 — Supplemental material. Download spectrum.02800-22-s0001.pdf, PDF file, 2.0 MB [file spectrum.02800-22-s0001.pdf]

Supplemental material

Table S1 The bulk soil and rhizosphere metabolome of *P. lactiflora*.

Table S2 The correlation of bacteria, fungi, and soil metabolome.

Table S3 Top 10 indicators of bacteria and fungi.

Table S4 Beta diversity comparisons (using Sørensen dissimilarity index) for bacterial and fungal communities of *P. lactiflora*.

Table S5 Mantel test of bacterial and fungal communities with environmental factors.

Table S6 Results of the dispersal-niche continuum.

Table S7 Results of the multiple regression analysis on matrices analysis (MRM) for each of the rhizosphere and bulk soil and the whole transect of *P. lactiflora.*

Table S8 Topological features at ASV level of the whole bacterial-fungal interkingdom networks and bipartite networks in bulk soil and rhizosphere.

Table S9 The content of root secondary metabolites of *P. lactiflora*.

Table S10 Spearman correlation of *P. lactiflora* root secondary metabolites of environmental factors.

Table S11 NCBI blast results for key ASVs

Table S12 Summary of soil sample information, sequencing, and statistical data of bacterial and fungal microbiome of *P. lactiflora*.

Table S13 The edaphic physicochemical properties of bulk soil at four origins of *Paeonia lactiflora*.

Figure S1 Soil metabolome profiles of the bulk soil and rhizosphere of *P. lactiflora*.

Figure S2 Relationships between bacterial and fungal communities of *P. lactiflora*.

Figure S3 Decomposing bacterial and fungal beta diversity to compare species replacement and richness differences in community composition.

Figure S4 Fit of the neutral community model (NCM).

Figure S5 The response of bacterial and fungal communities of *P. lactiflora* to environmental variables.

Figure S6 Identification of important environmental factors affecting bacteria and fungi in BS and RS.

Figure S7 Co-occurrence patterns of bacterial-fungal interkingdom bipartite networks.

Figure S8 Screening of environmental variables related to secondary metabolism of *P. lactiflora* root.

Figure S9 Microbial genera affecting paeoniflorin content.

Figure S10 Screening of key ASVs related to root secondary metabolism of *P. lactiflora*.

Figure S11 Identification of paeoniflorin-producing fungi by literature.

Supplemental results

2.1 Variations of soil metabolomics between BS and RS

The metabolic spectra of root-associated soils are made up of a diverse spectrum of compounds, which attract certain microbial to form complicated relationships with plants (1). The PCA analysis (Fig. 1B) revealed that the soil metabolic profiles in the RS differed from those in BS. However, PCA results did not allow complete separation of BS and RS soil metabolites from PC1 or PC2 axes. Permanova analysis indicated that both compartment (C) and origin (O) explained differences in soil metabolites similarly, 35.9% and 35.1%, respectively. From the above results, it can be found that the compartment has a greater impact on soil metabolism than the origin. The differential metabolites of BS and RS were further analyzed. The volcano map (Fig. S1A) and fold change results (Table S1) revealed that the abundances of most metabolites were greater in RS than in BS. Venn graph (Fig. S1B) illustrated that the two soil compartments share 82 metabolites, of which only the RS has 3 unique metabolites (e.g. 2,2-dimethyl-N-(2'-t-butylcarbonylphenyl)-Propanamide; succinic acid; quinolinic acid). Among them, only hexakis-O-(trimethylsilyl)-sedoheptulose were up-regulated ($logFC < -1$, $p < 0.05$) in BS, and the top 5 up-regulated ($logFC > 1$, $p < 0.05$) metabolites in RS were benzoic acid, D-threitol, 2,4,7,9-tetramethyldec-5-yne-4,7-diol, D-allose, and 1,2,3,4,5,6-hexa-O-trimethelsilyl-myo-inositol. Notably, BS and RS soil metabolites were not particularly different, probably because BS is also influenced by certain plants (samples were collected at 20 cm from the roots in a *P. lactiflora* farm field). This study's results

differed slightly from prior research on peppers, most likely as a result that the bulk soil was gathered 1 m away from the peppers (2). Additionally, soil microbial diversity may be influenced by chemical variety in root-associated soil (3).

The diversity (Shannon index) of the soil metabolites in the RS was significantly higher than that in BS (Fig. 1C). Similarly, soil metabolites' dissimilarity (Fig. 1D) in the RS was significantly higher than in the BS. Furthermore, the similarity of soil metabolites in the BS decreased significantly ($p < 0.01$) with increasing geographic distance (slope = –0.002), while the similarity did not change significantly ($p > 0.05$) in the rhizosphere (Fig. 1E). Mantel test (Fig. 1F) demonstrated that the fungal community had small but significant positive correlations ($p = 0.034$) with the soil metabolites (bray-curtis distance; $r = 0.159$). However, there were very few significant correlations between the soil metabolites and bacterial communities ($r = 0.094$, $p = 0.075$). In particular, the largest slope gradients in the linear regressions between soil metabolome and microbial communities appeared for the bacterial (slope = 0.254, $R^2 = 0.037$, $p < 0.001$) and fungal (slope = 0.286, $R^2 = 0.075$, $p < 0.001$) communities in RS.

2.2 Rhizosphere affects distribution and diversity of microbes in BS and RS

Bacteria and fungi in the same region have large differences in cell structure and ecological niche but are also closely related. Therefore, it is necessary to comprehensively understand the relationship between bacteria and fungi in BS and RS. Since the diversity of bacteria is much higher than that of fungi in most circumstances, the ratio of bacterial-to-fungal diversity was determined to explore the

equilibrium between bacterial and fungal diversity (4). In this study, the ratio of bacterial/fungal α-diversity showed that the equilibrium of BS was slightly but not significantly higher than that of RS. The linear regression and spearman correlation analysis (Fig. 3B) showed that the α-diversity of bacteria and fungi in BS had a significantly negative correlation, while RS had a positive correlation. The mantel test and procrustes analysis based on bray-curtis dissimilarity measures were used to investigate the link between bacterial and fungal communities. As shown in Fig. S2B, the bacterial community showed a significant positive correlation with the fungal community both in the BS and RS. In addition, the bacterial community revealed a significant correlation with the fungal community ($p < 0.001$), with the Procrustes sum of squares (M^2) 0.765 (Fig. S2D). Notably, the findings of the mantel test revealed that bacterial alpha diversity was strongly positively linked with fungal community dissimilarity (Table S5).

2.3 Community assembly was governed by stochastic processes primarily belonging to dispersal limitation

However, the results of the Modified Stochastic Ratio (MST) indicated the dominance of deterministic processes in the communities of both BS and RS. (Fig. 4C), which is inconsistent with the results of βNTI. The phylogenetic tree data were considered in the calculation of βNTI. In contrast, MST is an expansion based on the beta dissimilarity index, which reflects the contribution of random assembly relative to deterministic assembly (5). The different principles of the two calculation methods and the size of the samples may be the reasons for the inconsistent results.

Whether dispersal or ecological niche processes dominate community construction in the dispersal-niche continuum can be assessed by the dispersal-niche continuum index (DNCI) (6). In this study, the DNCI results (Table S6) indicated that dispersal processes were the main driver of bacterial (DNCI = −147.8485±9.145781) and fungal (DNCI = −62.01647±5.56185) community construction. The negative DNCI values revealed that dispersion assembly was the primary mechanism shaping the analyzed site units consisting of bacterial and fungal communities. Simultaneously, the absolute value of the DNCI of the bacterial community was greater than that of the fungus community, suggesting that the bacterial community's diffusion dominance process was more intense than that of the fungal community. In contrast to other natural habitats, the formation of microbial communities in agricultural soils was more controlled by neutral (e.g., stochastic) processes, suggesting that long-term agriculture and human-managed activities may promote the stochastic influx and dispersal of microorganisms (7).

Deterministic processes include the selection of abiotic environmental factors (environmental filtering) and the mutual antagonism and synergy between species. Furthermore, soil physicochemical characteristics such as pH, wetness, and soil characteristics can influence soil bacterial and fungal communities and, as a result, root-associated microbial communities(8). The environmental variables that were strongly linked with β-diversity were first filtered using a mantel test to further investigate the interaction between microbial communities and environmental variables (Table S5). Multiple regression on distance matrices (Table S7) was used to

further identify the relative contributions of environmental variables to bacterial (BS: 67% and RS: 17%) and fungal community similarity (BS: 73% and RS: 69%). The dominating factor determining the bacterial communities in each compartment, according to the MRM, were srad (BS) and altitude (RS), respectively. The MRM revealed that latitude (BS) and SOM (RS) were the primary drivers for the fungal communities in BS and RS, respectively. CCA analysis (Fig. S5A) was performed based on the following screened environmental factors, and the results revealed that cation exchange capacity (CEC), soil water content (SWC), soil texture (0.05~0.002mm, ST3), alpha diversity of bacteria (B_Chao1), the composition of bacterial communities (adjusted principal coordinate analysis aPCoA1, B_apcoa1), and latitude had significant contributions to bacterial communities, while CEC, pH, B_Chao1, NH_4^+-N, and B_apcoa1 had a major impact on fungal communities. Interestingly, both bacterial α-diversity and β-diversity had massive influences on fungal communities. Laboratory experiments have demonstrated that fungi affect the diversity and composition of bacterial communities (7). Hierarchical partitioning analysis (Fig. S5B) indicated that CEC had the highest explanatory rate for bacterial and fungal communities. Moreover, the PCoA diagrams (Fig. S6) highlighted the pattern of distribution of microbial community structures with important environmental factors, and the findings indicated that the -diversity varied considerably with the CEC. The size of the CEC can be used as an index to evaluate the ability of soil to retain fertilizer(9). However, the present state of study on the influence of CEC on the microbes is uncertain, and further experimental investigations are required. Notably,

the results of the hierarchical partitioning indicated that environmental variables had a quite low interpretation rate (bacteria: 6.3%; fungi: 8.1%) and a large level of the unexplained component (bacteria: 93.7%; fungi: 91.9%). Correspondingly, it is speculated that the deterministic process is dominated by species-to-species interactions.

2.5 Integrated modulations of P. lactiflora root secondary metabolites.

Secondary metabolites (SMs) of *P. lactiflora* (Table S9) are the material basis for their clinical efficacy and an important component of *P. lactiflora* defense against pathogenic attack and environmental stress(10). Abiotic and biotic environments that are constantly changing have an impact on the appropriate synthesis and accumulation of SMs, which are tightly regulated in both space and time(11). The *P. lactiflora* root secondary metabolites were concurrently influenced by both abiotic ((location, soil, and climate) and biotic (diversity and composition of microbe) variables. Initially, spearman correlation analysis (Fig. 6A and Table S10) was used to filter environmental variables that were significantly correlated with root secondary metabolites. Importantly, only the composition of fungi (F_apcoa1) in RS was significantly negative with benzoyloxypeoniflorin, ethyl gallate, and oxypaeoniflora. For abiotic factors, soil water content (SWC), longitude, and soil pH were significantly positively correlated with SMs of *P. lactiflora* (Fig. S8A). Taking the most representative paeoniflorin in paeoniflorin as an example, linear regression analysis showed that paeoniflorin was significantly positively correlated with altitude, SWC, pH, and MBC, and significantly negatively correlated with mean annual precipitation

(MAP) (Fig. S8C). The MAP distribution of the sampling point map was also consistent with the content of paeoniflorin (Fig. S8B). Although several studies have shown that water stress, or drought stress, can promote the content of plant secondary metabolism (12), this research shows that the abundance of paeoniflorin is directly proportional to soil moisture. This indicates that *P. lactiflora* may prefer a moist soil environment, which provides theoretical advice for the cultivation of *P. lactiflora*.

The random forest regression model analysis was performed to identify microbial genera affecting paeoniflorin. For the bacterial genus of BS, the overall model explanation was 85.13% ($p < 0.001$), *Rhodoplanes* and *Pesudoxanthomonas* had a significant effect on paeoniflorin, and the regression fitting curve showed that its abundance was positively correlated with the content of paeoniflorin (Fig. S9A). The explanation for the bacterial genus in RS was 43.54% ($p < 0.001$), *Erwinia* and *Sphingobium* had a significant influence on paeoniflorin. Similarly, their abundance was positively correlated with paeoniflorin content. (Fig. S9B). On the contrary, in BS (74.84%, $p < 0.001$) and RS (68.7%, $p < 0.001$), the significantly related fungal genera were *Apiotrichum* (BS), *Solicoccozyma* (BS), *Alternaria* (RS), *Vishniacozyma* (RS), and their abundance correlation with the content of paeoniflorin increased first and then decreased (Fig. S9C and D).

Reference

1. Kuzyakov Y, Razavi BS. 2019. Rhizosphere size and shape: temporal dynamics and spatial stationarity. Soil Biology and Biochemistry 135:343-360.
2. Song Y, Li X, Yao S, Yang X, Jiang X. 2020. Correlations between soil metabolomics and bacterial

community structures in the pepper rhizosphere under plastic greenhouse cultivation. Science of The Total Environment 728:138439.

3. Baran R, Brodie EL, Mayberry-Lewis J, Hummel E, Da Rocha UN, Chakraborty R, Bowen BP, Karaoz U, Cadillo-Quiroz H, Garcia-Pichel F. 2015. Exometabolite niche partitioning among sympatric soil bacteria. Nature communications 6:1-9.
4. Li M, Dai B, Tang Y, Lei L, Li N, Liu C, Ge T, Zhang L, Xu Y, Hu Y. 2019. Altered bacterial-fungal interkingdom networks in the guts of ankylosing spondylitis patients. Msystems 4:e00176-18.
5. Ning D, Deng Y, Tiedje JM, Zhou J. 2019. A general framework for quantitatively assessing ecological stochasticity. Proc Natl Acad Sci U S A 116:16892-16898.
6. Vilmi A, Gibert C, Escarguel G, Happonen K, Heino J, Jamoneau A, Passy SI, Picazo F, Soininen J, Tison‐Rosebery J. 2021. Dispersal–niche continuum index: a new quantitative metric for assessing the relative importance of dispersal versus niche processes in community assembly. Ecography 44:370-379.
7. Jiao S, Peng Z, Qi J, Gao J, Wei G. 2021. Linking bacterial-fungal relationships to microbial diversity and soil nutrient cycling. Msystems 6:e01052-20.
8. Bai B, Liu W, Qiu X, Zhang J, Zhang J, Bai Y. 2022. The root microbiome: Community assembly and its contributions to plant fitness. Journal of Integrative Plant Biology 64:230-243.
9. Heiniger RW, McBride RG, Clay DE. 2003. Using soil electrical conductivity to improve nutrient management. Agronomy Journal 95:508-519.
10. Li Y, Kong D, Fu Y, Sussman MR, Wu H. 2020. The effect of developmental and environmental factors on secondary metabolites in medicinal plants. Plant Physiology and Biochemistry 148:80-89.
11. Verma N, Shukla S. 2015. Impact of various factors responsible for fluctuation in plant secondary metabolites. Journal of Applied Research on Medicinal and Aromatic Plants 2:105-113.
12. Zobayed S, Afreen F, Kozai T. 2007. Phytochemical and physiological changes in the leaves of St. John's wort plants under a water stress condition. Environmental and Experimental Botany 59:109-116.

Supplementary Figures

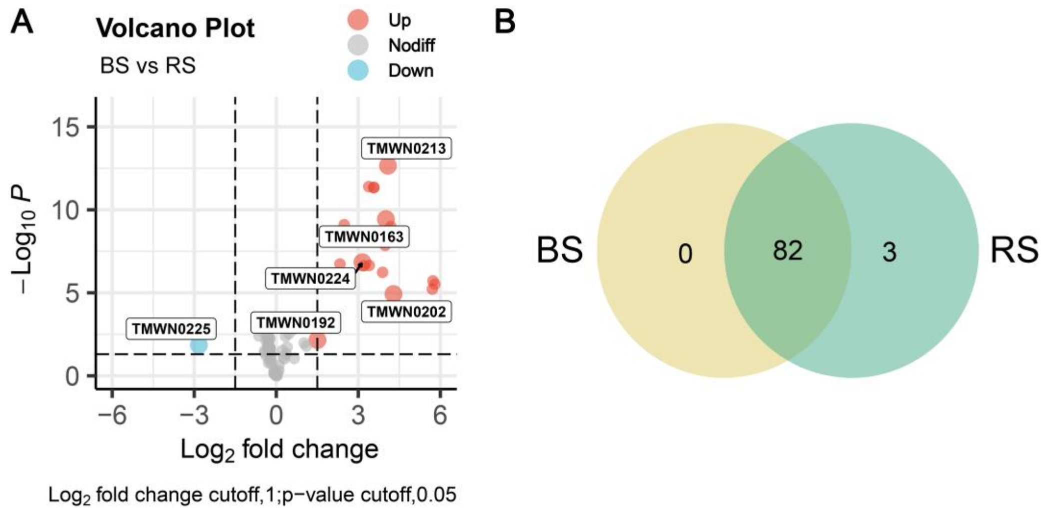

Figure S1 Soil metabolome profiles of the bulk soil (BS) and rhizosphere (RS) of *Paeonia lactiflora*. (A) Volcano map of the soil metabolome in the BS and RS. The blue dots represent downregulated differentially soil metabolites, the red dots represent upregulated differentially soil metabolites, and the gray dots represent metabolites detected but not significantly different. (B) Venn graph indicates the number of soil metabolites in each group and the number of common metabolites.

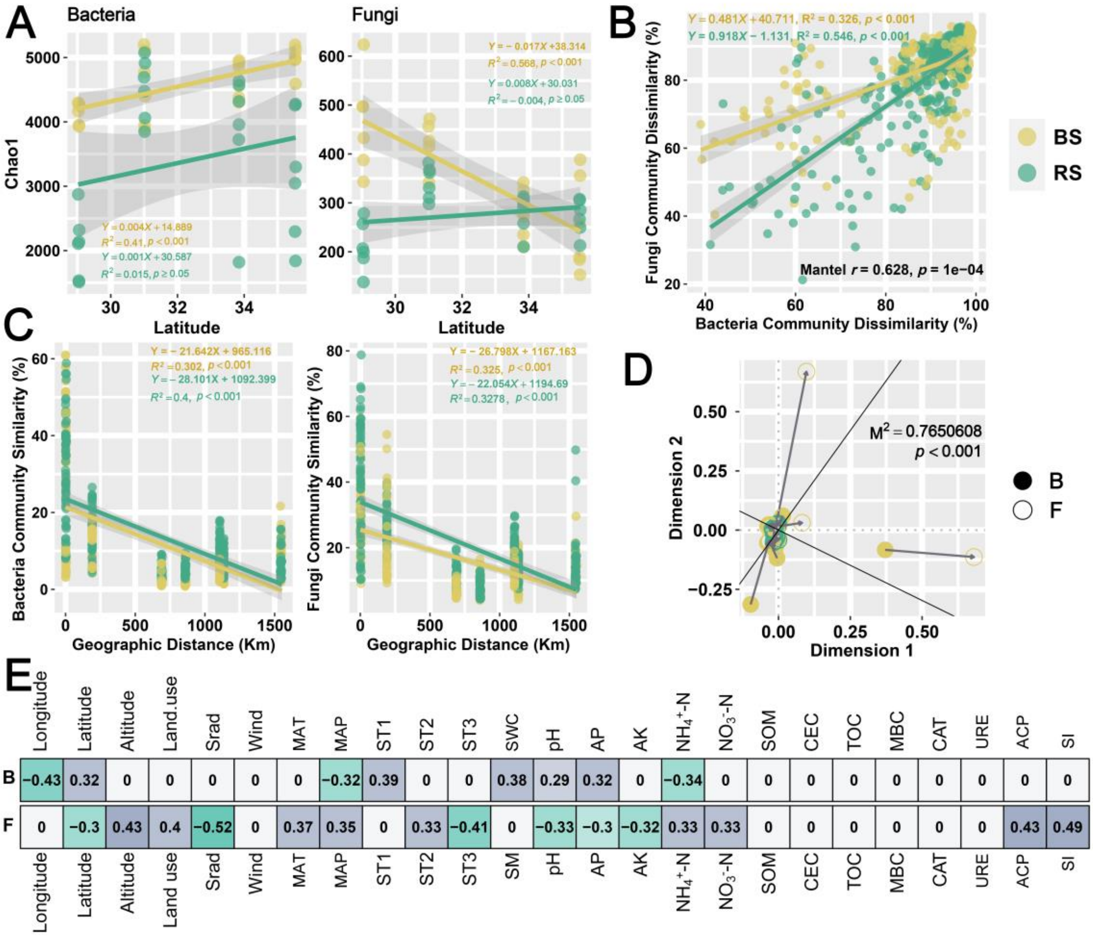

Figure S2 Relationships between bacterial and fungal communities of *P. lactiflora*. (A) Latitude diversity pattern of bacteria and fungi. (B) Correlations between bacterial community similarity and the fungal community dissimilarity in the bulk soil and rhizosphere soil are determined using the Mantel test. The correlation coefficients (*r*) and significance (*p*-values) are provided. The *p*-values are derived from one-tailed tests based on 999 permutations. The rate of bacterial community turnover (slopes) with the variation in fungal community, and the fitting degree (R^2) and significance (*p*-values) of the linear fitting model are provided. The shaded region represents the 95% confidence limits of the regression estimates. (C) Distance-decay relationships (DDR) between microbial community and geographical distance. (D) Procrustes analysis of the correlation between bacterial and fungal communities based on the NMDS (Bray-Curtis) (999 permutations). (E) Correlation between bacterial and fungal alpha diversity and environmental variables. The number in the square is obtained by multiplying the spearman correlation (ρ) and the significance (p). By default, the number $p > 0.05$ is classified as 0. BS: bulk soil, RS: rhizosphere soil. B: Bacteria; F: Fungi.

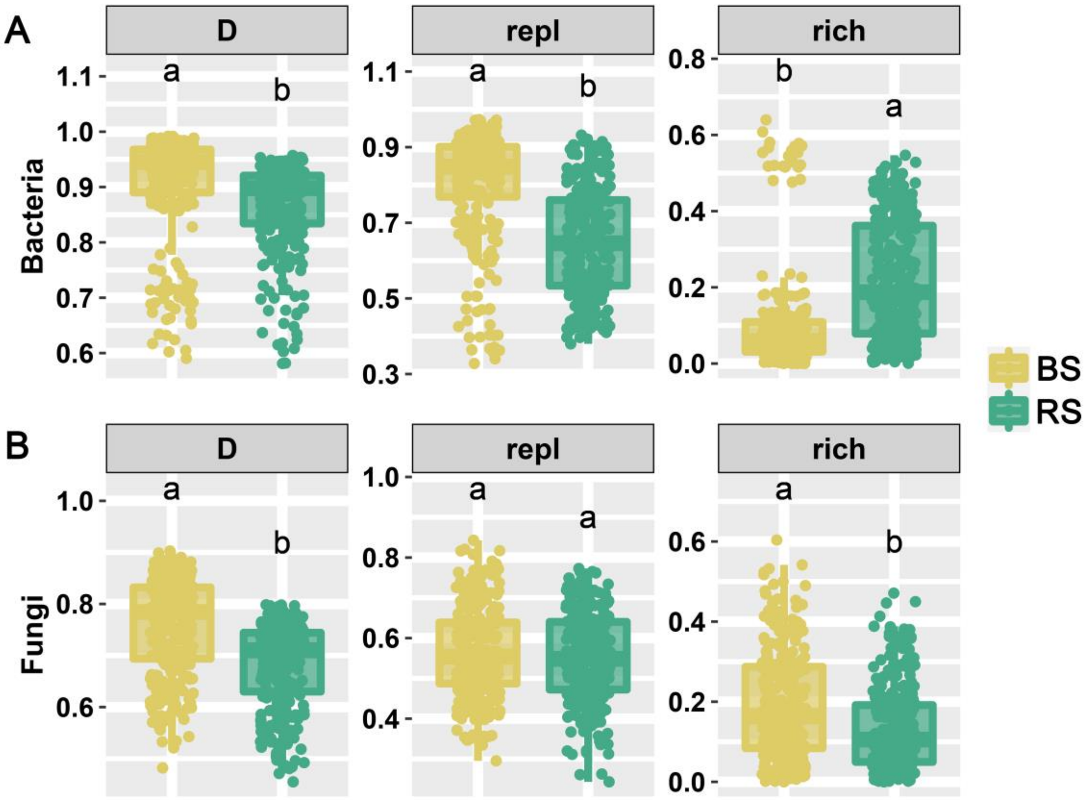

Figure S3 Decomposing bacterial (A) and fungal (B) beta diversity to compare species replacement and richness differences in community composition. D: dissimilarity index between sample pairs; repl: replacement between sample pairs; rich: richness difference between sample pairs. BS: bulk soil, RS: rhizosphere soil.

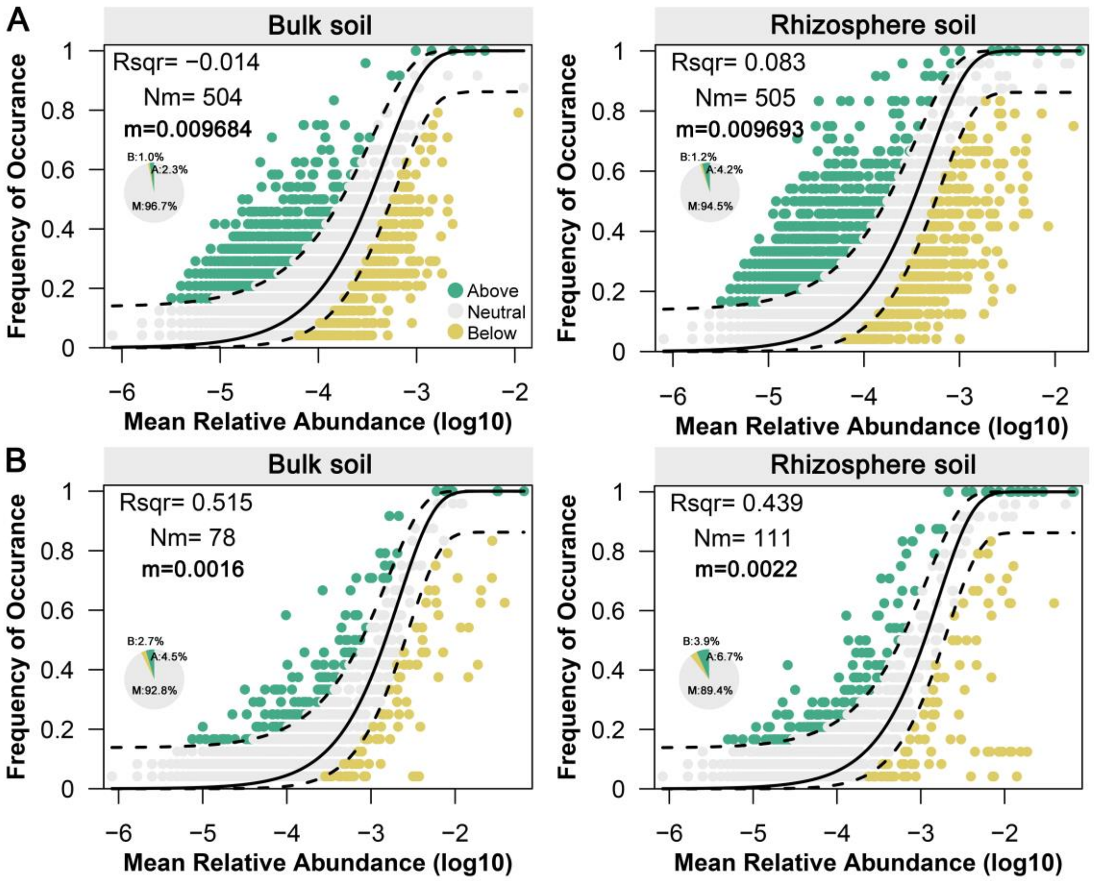

Figure S4 Fit of the neutral community model (NCM) of bacterial (A) and fungal (B) community assembly. R^2 (Rsqr) indicates the fit to this model. Nm is the product of metacommunity size (N) and migration rate (m), which quantifies the estimate of dispersal between communities and determines the correlation between frequency of occurrence and relative regional abundance. The solid line indicates the best fit to the NCM, and the dashed lines represent 95% confidence intervals around the model prediction. The pie charts depict the ratio of above taxa (ASVs that occur more frequently than predicted by the model are shown in green), neutral taxa (ASVs that occur within prediction are shown in grey) and below taxa (ASVs that occur less frequently than predicted are shown in yellow).

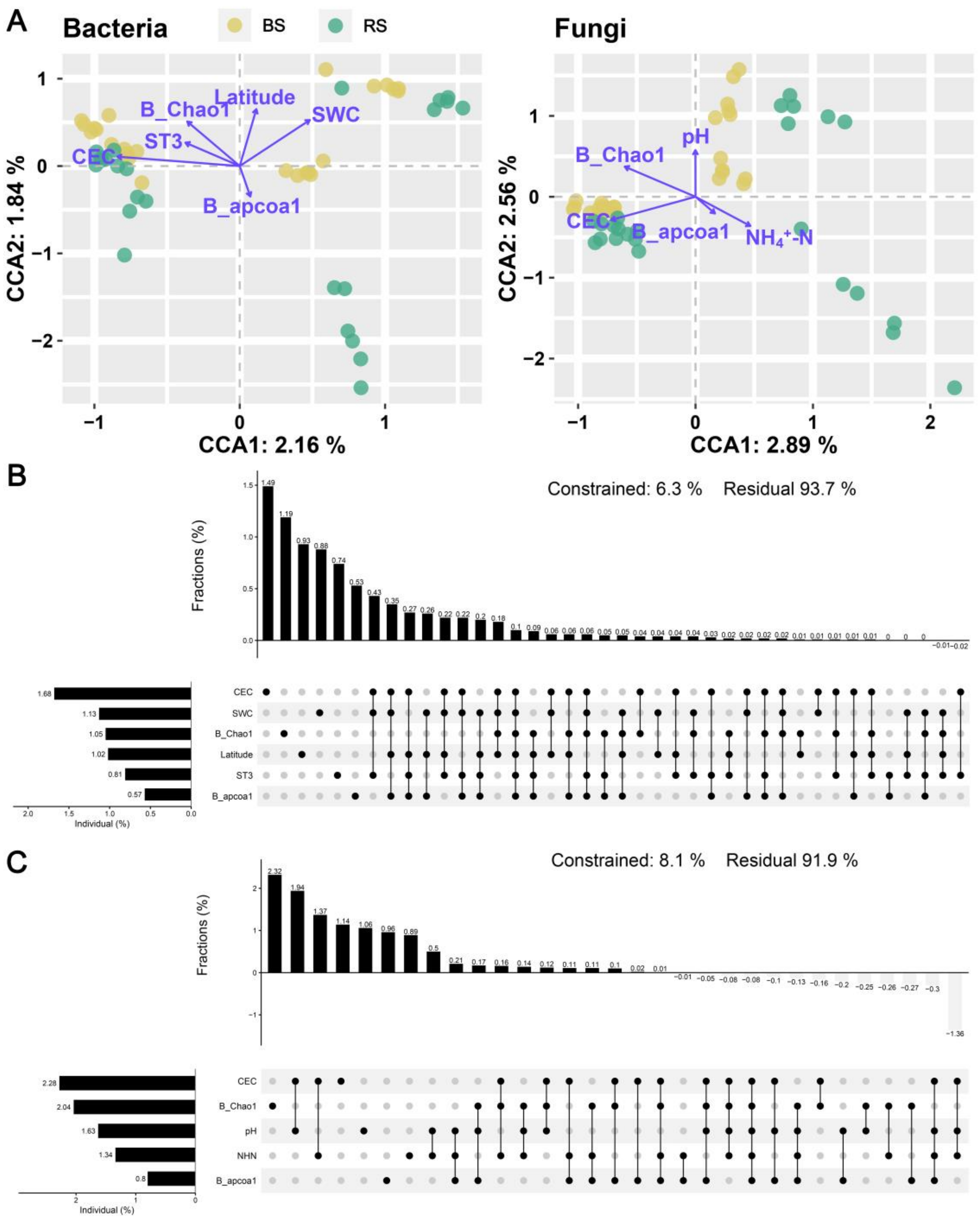

Figure S5 The response of bacterial and fungal communities of *P. lactiflora* to environmental variables. (A) Canonical correspondence analysis (CCA) is used to evaluate the effect of environmental variables on the bacterial and fungal community composition. The total variance (in percent) explained by each axis is indicated in parentheses. Environmental variables significantly explained the observed compositional variation in the bacterial and fungal community (permutational ANOVA for CCA). Hierarchical partitioning to obtain canonical analysis of relative importance of explanatory variables. Residuals represent unexplained changes in the microbial community. (B): Bacteria; (C): Fungi. BS: bulk soil, RS: rhizosphere soil. B_Chao1: alpha diversity of bacteria; B_apcoa1: composition of bacterial communities (adjusted

principal coordinate analysis aPCoA1)

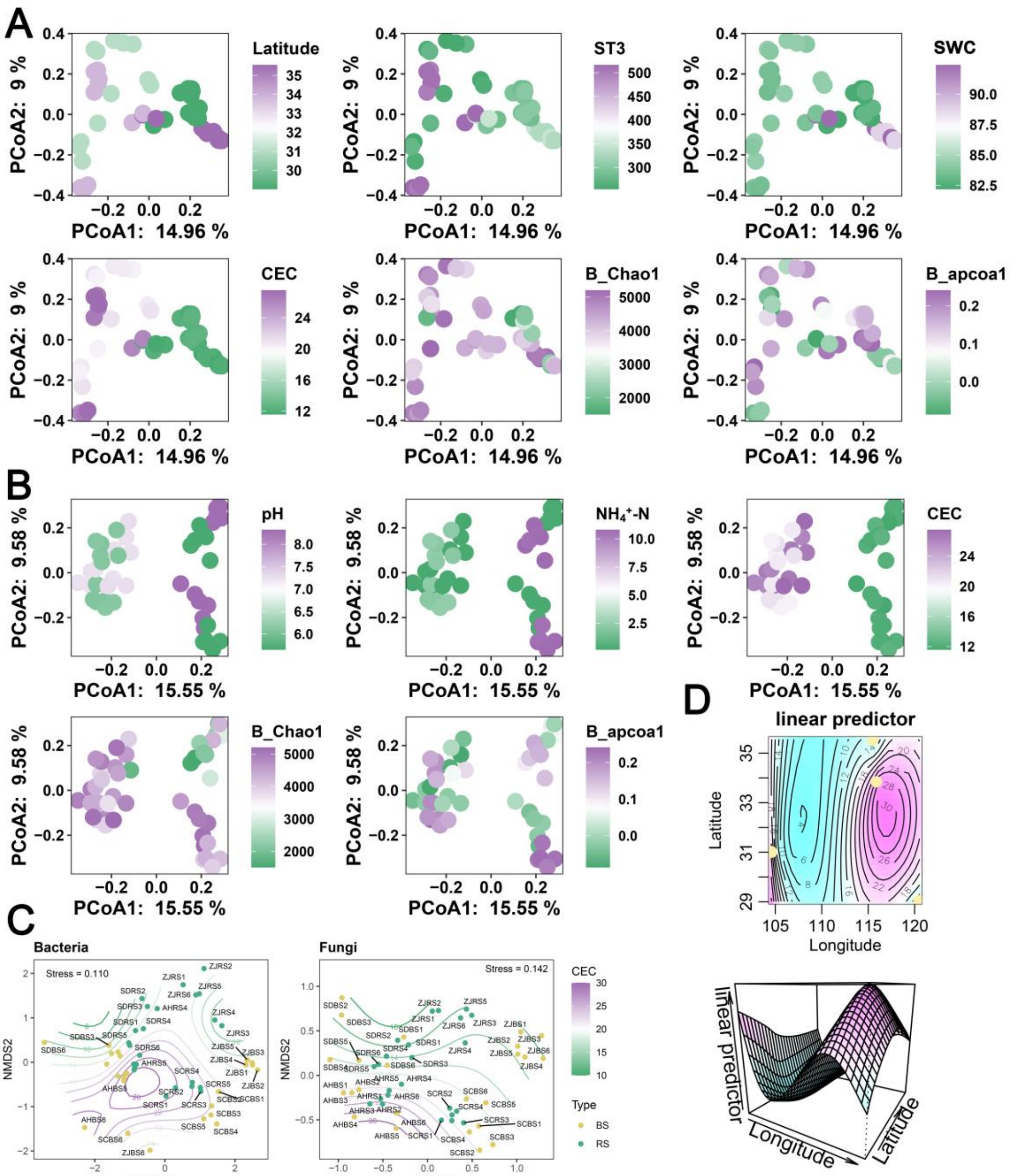

Figure S6 Identification of important environmental factors affecting bacteria and fungi. PCoA diagram shows the distribution pattern of bacterial (A) and fungal (B) community structures with environmental variables. (C) Non-metric multidimensional scaling (NMDS) of smooth regression shows the relationship between community heterogeneity and cation exchange capacity (CEC). (D) The smooth regression analysis of the cation exchange capacity (CEC) fitted by the additive model with the change of the latitude and longitude geographic variables. The yellow dots represent the distribution of microbial samples. Top: contour map; Bottom: 3D map.

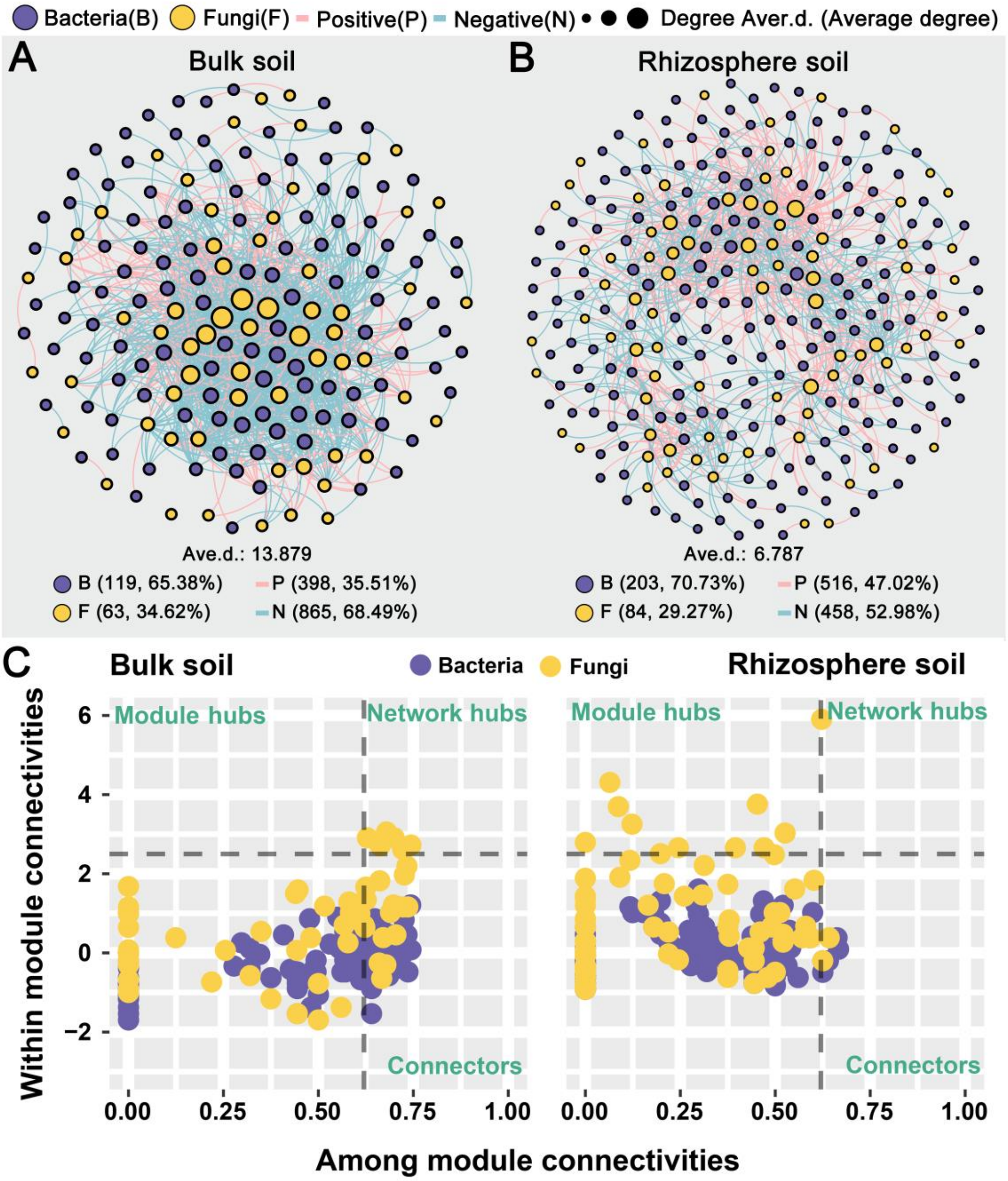

Figure S7 Co-occurrence patterns of bacterial-fungal interkingdom bipartite networks in the bulk soil (A) and rhizosphere soil (B) of *P. lactiflora*. (C) Z_i-P_i (Z_i: within-module connectivity and P_i: among module connectivity) plot of ASVs. Module hubs: highly connected nodes within modules, $Z_i > 2.5$ and $P_i < 0.62$; Connectors: connect nodes, nodes with high connectivity between two modules, $Z_i < 2.5$ and $P_i > 0.62$; Network hubs: network hubs, nodes with high connectivity in the entire network, $Z_i > 2.5$ and $P_i > 0.62$.

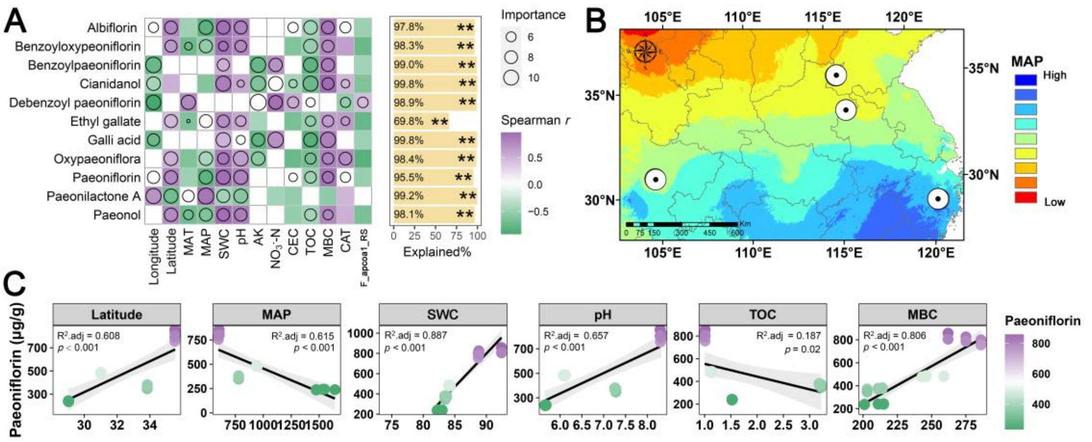

Figure S8 Screening of environmental variables related to secondary metabolism of *P. lactiflora* root. (A) The Spearman correlation and random forest analyses of the environmental variables and root secondary metabolites. Significant relationships ($p < 0.05$) are indicated by circles (random forest analysis). The size of the circle indicates the random forest mean predictor importance of the environmental variables for the root secondary metabolites. The histograms indicate the total explained variation degree of the root secondary metabolites (response variables) by environmental variables as a whole (explanatory variables) based on random forest analysis. The R^2 and significance of the full model are calculated by the A3 R package. (B) Mean annual precipitation map of the sampling sites. (C) Linear regression analysis of paeoniflorin and environmental variables.

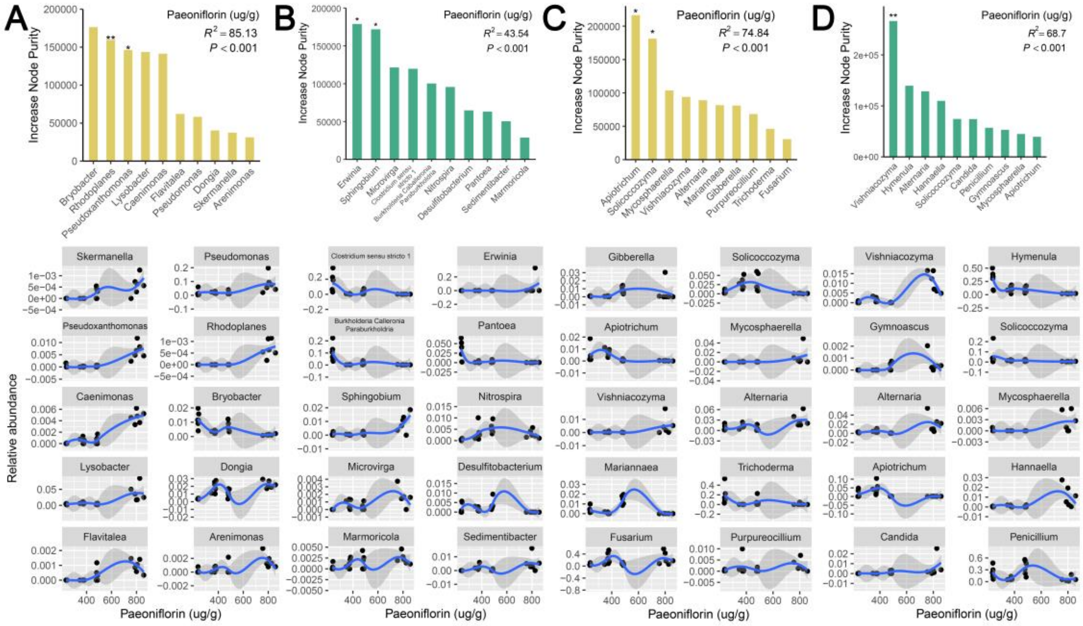

Figure S9 Microbial genera affecting paeoniflorin content.Top: Contribution of the top 10 key genera to the paeoniflorin based on random forest regression models. Bottom: Regression relationship between top 10 key genera and paeoniflorin. (A) Bacteria in bulk soil; (B) Bacteria in rhizosphere soil; (C) Fungi in bulk soil; (D) Fungi in rhizosphere soil.

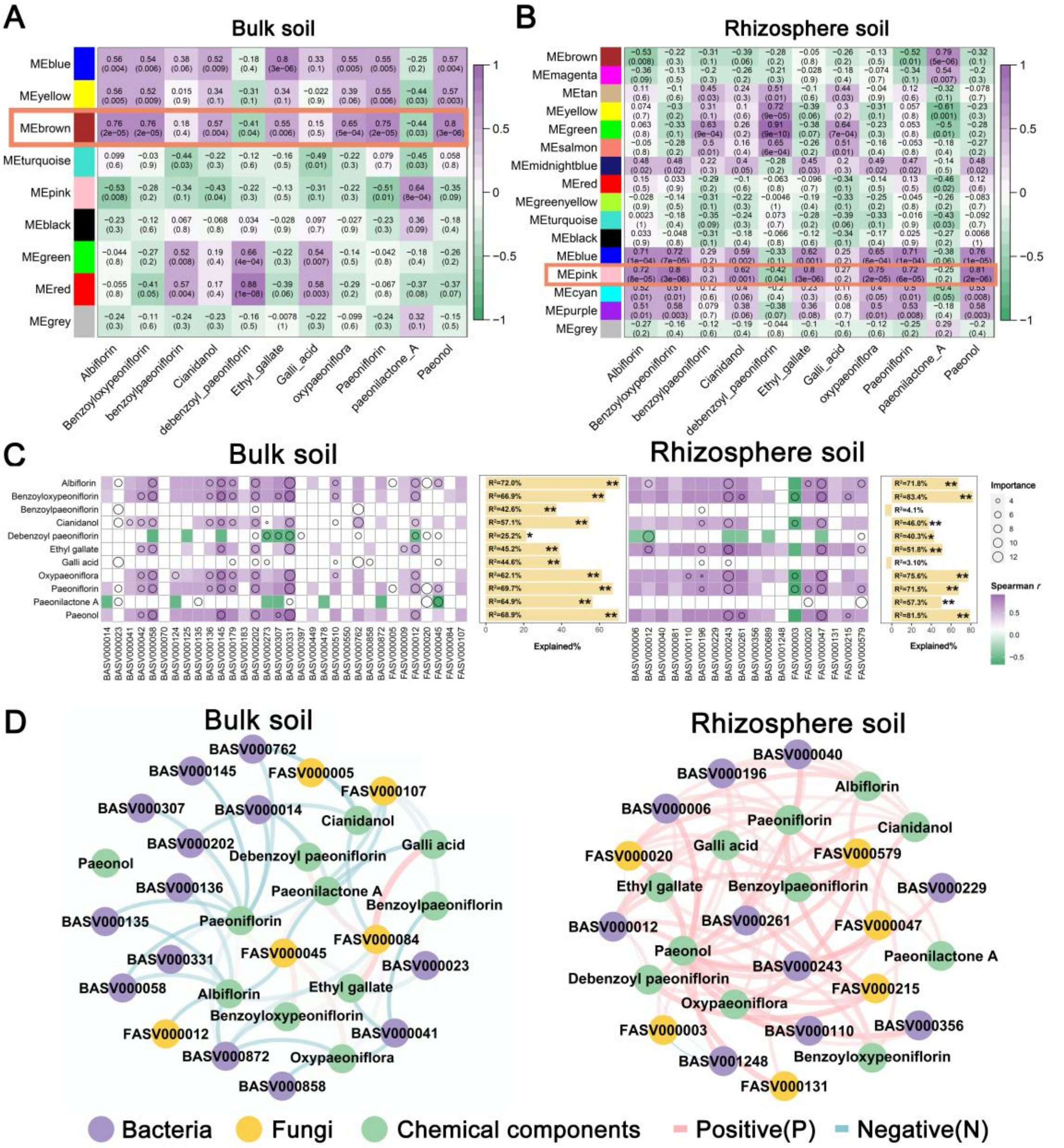

Figure S10 Screening of key ASVs related to root secondary metabolism of *P. lactiflora*. (A) and (B) WGCNA analysis of root secondary metabolites and microbial community abundance. (C) The Spearman correlation and random forest analyses of the environmental variables and root secondary metabolites. Significant relationships ($p < 0.05$) are indicated by circles (random forest analysis). The size of the circle indicates the random forest mean predictor importance of the environmental variables for the root secondary metabolites. The histograms indicate the total explained variation degree of the root secondary metabolites (response variables) by environmental variables as a whole (explanatory variables) based on random forest analysis. The R^2 and significance of the full model are calculated by the A3 R package. (D) Co-occurrence network of key modules. Bulk soil: brown modules; Rhizosphere soil: pink modules.

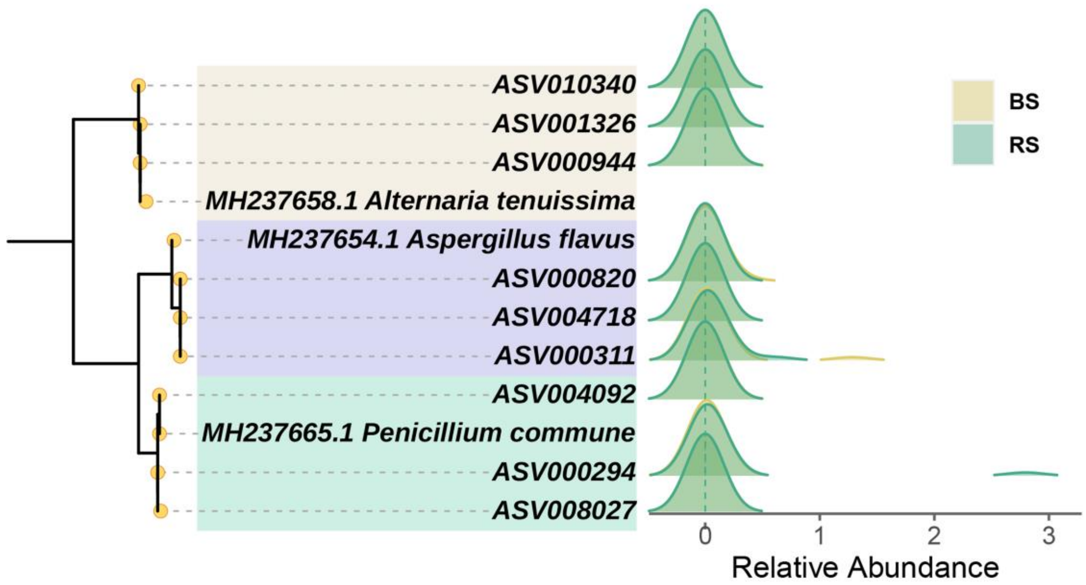

Figure S11 Identification of paeoniflorin-producing fungi by literature. Ridgeline plots show the relative abundance of key ASVs in the bulk soil and rhizosphere soil. The key ASVs and the sequences obtained from the blast results on NCBI are constructed using the maximum likelihood method to construct a phylogenetic tree.
